# Experimental and theoretical evidences for the formation of transition metal complexes with five coplanar metal–carbon σ bonds

**DOI:** 10.1093/nsr/nwad325

**Published:** 2023-12-20

**Authors:** Yuhui Hua, Ming Luo, Zhengyu Lu, Hong Zhang, Dafa Chen, Haiping Xia

**Affiliations:** Shenzhen Grubbs Institute and Guangdong Provincial Key Laboratory of Catalysis, Department of Chemistry, Southern University of Science and Technology, Shenzhen 518055, China; Shenzhen Grubbs Institute and Guangdong Provincial Key Laboratory of Catalysis, Department of Chemistry, Southern University of Science and Technology, Shenzhen 518055, China; Shenzhen Grubbs Institute and Guangdong Provincial Key Laboratory of Catalysis, Department of Chemistry, Southern University of Science and Technology, Shenzhen 518055, China; Department of Chemistry, College of Chemistry and Chemical Engineering, Xiamen University, Xiamen 361005, China; Shenzhen Grubbs Institute and Guangdong Provincial Key Laboratory of Catalysis, Department of Chemistry, Southern University of Science and Technology, Shenzhen 518055, China; Shenzhen Grubbs Institute and Guangdong Provincial Key Laboratory of Catalysis, Department of Chemistry, Southern University of Science and Technology, Shenzhen 518055, China

**Keywords:** M–C bond, polydentate ligands, metallaaromatics, DFT calculations, aromaticity

## Abstract

The *σ* bond is an important concept in chemistry, and the metal–carbon (M–C) *σ* bond in particular is a central feature in organometallic chemistry. Synthesis of stable complexes with five coplanar M–C *σ* bonds is challenging. Here, we describe the synthesis of two different types of stable complexes with five coplanar M–C *σ* bonds, and examine the stability of such complexes which use rigid conjugated carbon chains to chelate with the metal center. Density functional theory (DFT) calculations show that the M–C *σ* bonds in these complexes have primarily a covalent character. Besides the *σ* nature, there are also a *π* conjugation component among the metal center and carbons, which causes delocalization. This work expanded the coplanar M–C *σ* bonds to five.

## Teaser

Two different types of all-carbon-surrounded transition metal complexes with five coplanar M–C *σ* bonds were produced, and they show good stability in air and moisture.

## INTRODUCTION

M–C σ bonds are one of the core features of organometallic complexes. As special organometallics, metallaaromatics can be defined as aromatic complexes that have one or more metal atoms in their aromatic ring [1–9]. Metallaaromatics mainly consist of metallabenzenes [10–16], metallabenzynes [17–20], heterometallaaromatics [21–24], dianion metalloles [25,26], spiro metalloles [27–29] and carbolong complexes [30,31], all containing at least one M–C *σ* bond. Specifically, carbolong complexes, which include metallapentalynes, metallapentalenes, and their derivatives, have not less than three M–C *σ* bonds. The name ‘carbolong’ comes from the fact that both metallapentalynes and metallapentalenes contain a long carbon chain (≥7C) coordinated to a bridgehead metal, and interestingly, the three M–C *σ* bonds within the metallapentalyne and metallapentalene rings are in the same plane.

**Figure 1.**
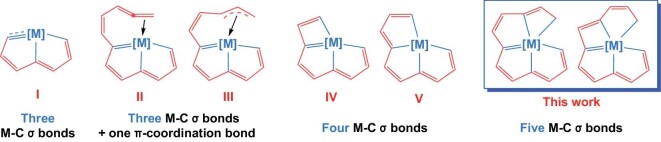
Typical previous carbolong skeletons with three or four coplanar M–C *σ* bonds (**I–V**) and the carbolong skeletons with five coplanar M–C *σ* bonds in this study.

The first carbolong complexes were metallapentalynes containing three coplanar M–C *σ* bonds (Fig. 1, **I**), which were reported by our group in 2013 [32]. Thereafter many other carbolong complexes that contain three coplanar M–C *σ* bonds as well, such as metallapentalenes with skeletal structure **I** [33,34], and their derivatives with **II** [35] and **III** [36] frameworks, were discovered (Fig. 1). Besides, some carbolong complexes with four coplanar M–C *σ* bonds, for instance, metallapentalene derivatives with **IV** [37,38] and **V** [39] structures, have also been reported (Fig. 1). These structurally unique complexes exhibit interesting properties, and have been applied in several areas [30].

As our interest in carbolong chemistry continues, we aimed to prepare substances with more coplanar M–C *σ* bonds. Herein, we report the preparation and characterization of two types of osmium complexes containing five coplanar M–C *σ* bonds. The difference between the two types is the size and order of the rings in their structures. Density functional theory (DFT) calculations were performed to characterize the bonding in these unique structures. The successful construction of these structures may be attributed to the use of a carbon chain as a rigid and polydentate ligand (Fig. 1) and the conjugation effect between *sp^2^*carbons and the metal center, which maintains the M–C *σ* bonds in a single plane and prevents elimination of the two neighboring carbons that bind to the metal. These organometallics were found to be stable at temperatures up to 100°C in moisture or air.

## RESULTS AND DISCUSSION

### Design, synthesis, characterization and stability of complexes with five coplanar Os–C σ bonds

**Figure 2.**
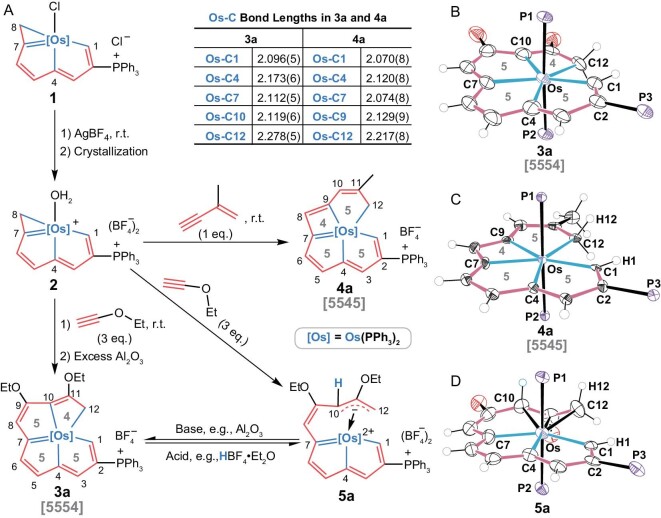
Synthesis, structures and reactivity of the prepared [5554] and [5545] complexes (the numbers in the rings are the ring sizes). (A) Synthesis and Os–C bond lengths in the [5554]-type complex (**3a**) and the [5545]-type complex (**4a**) and the reversible reaction between **3a** and its *η^3^*-coordinated intermediate (**5a**). X-ray crystallographic structures for cations of **3a** (B), **4a** (C) and **5a** (D) are with 50% probability (the phenyl groups in the PPh_3_ moieties and ethyl groups on oxygen have been omitted for clarity).

Complex **1** (Fig. 2A), which has been reported to possess possibilities for continued reaction to form a higher conjugated system [35,36,39], was chosen as the starting material. We used AgBF_4_ to remove the chloride ligand from the osmium center of complex **1**, resulting in the isolation of complex **2** (Fig. 2A), whose structure was confirmed by X-ray crystallographic analysis (Fig. S1). Subsequently, materials with unsaturated carbons were used to construct rigid and conjugated systems, so that the corresponding M–C *σ* bonds were confined in a plane to improve their stability. Complex **2** was treated first with ethyl ethoxyethyne (HC≡COEt) and then with excess neutral alumina to absorb the acid that was generated, producing complex **3a**. The structure of **3a** is denoted as [5554], indicating the size of the rings, recorded in the direction from C1 to C12. An *η^3^*-coordinated intermediate (**5a**) with the skeleton structure **III** as shown in Fig. 1 was also isolated in the absence of Al_2_O_3_. Treatment of **5a** with a base such as Al_2_O_3_ was found to yield **3a** reversibly (Fig. 2A). Complex **2** was also treated with 2-methyl-1-butene-3-yne (HC≡CCMe=CH_2_) and produced complex **4a**, whose structure is denoted [5545] (Fig. 2A). Similar structures with different substituents (**3b, 4b–4d**) could also be obtained from different substrates (see details of the synthesis in the Supplementary Materials).

To investigate the structures of **3a** and **4a**, X-ray crystallographic analysis was performed (Fig. 2B and 2C). The skeletons of both of these structures contain one metal center and a multidentate hydrocarbon chain. The mean deviations from the least-squares plane of C1–C12 and Os were determined to be 0.160 Å in **3a** and 0.036 Å in **4a**, indicating good planarity of both compounds. The bond lengths of the five M–C *σ* bonds in **3a** and **4a** were determined, and are shown in Fig. 2A. The Os–C12 bonds in both **3a** and **4a** were found to be longer than the other Os–C bonds because the C12 atom is *sp^3^* hybridized, whereas carbons C1–C11 are all *sp^2^* hybridized and conjugated with osmium. In addition to the planarity of the skeletons, the X-ray data show five coplanar M–C bonds in each structure. The intermediate **5a** was also characterized by X-ray diffraction and its structure is shown in Fig. 2D. It is a non-planar structure because it contains an *η^3^*-coordination fragment.

In the X-ray crystallographic data, average distances between H1 and the protons on C12 (two H12 atoms) were determined to be 2.635 Å in **3a** and 2.394 Å in **4a**, which prompted us to consider the possibility of adding one more Os–C *σ* bond in the plane. In **3a** and **4a**, the carbon chain ligands composed of 12 carbons form 5 coplanar *σ* bonds with the metal, which significantly diminishes the space available to the substituents on the terminal carbons C1 and C12 raising the possibility of repulsion between the substituents on C1 and C12. The heteronuclear multiple bond correlation (HMBC) spectra of **3a** and **4a** (Figs S2 and S3, respectively) reveal strong interactions between C1 and H12 and between C12 and H1, which may be a result of the crowding of these atom pairs. This strong bond correlation appears to depend on repulsive forces and this was further confirmed by analysis of the noncovalent interactions (NCIs) derived from the DFT calculations (Figs S4 and S5) [40]. This suggests a steric limit on the number of carbons in the equatorial plane around the osmium center, which may not support the introduction of one extra equatorial Os–C *σ* bond in these two systems.

Thermal stability studies of **3a** and **4a** were then performed to investigate the stabilities of their skeletons (Table S1). These experiments showed that **4a** is stable in air for 1 month at room temperature (purity >95%) and that **3a** has no detectable decomposition even after more than 6 months. Complex **3a** was also found to be stable in air for at least 1 day at 100°C in the solid state with purity >95%. The stability of **3a** and **4a** can be attributed to their structures: the conjugated and rigid polydentate systems assume stable configurations, in which five covalent M–C bonds tightly connect the metal and the organic moiety. In addition, a chemical reagent tolerance was also performed on **3a** and **4a**. Complex **3a** can tolerate extreme conditions and reactants such as sodium (Na), oxidants such as hydrogen peroxide (H_2_O_2_) and bases such as sodium hydride (NaH). In acidic media, however, **3a** is converted to **5a**. This conversion is reversible, and **3a** can be recovered after removal of the acid (Fig. 2A). In contrast, compound **4a** appears to be less capable of tolerating a strong chemical environment than **3a**. This may be partly due to the strain in the four-membered ring of **4a**.

On the other hand, the stability of **3a** and **4a** may also be associated with their aromaticity [41]. The aromaticity of the skeletons of both **3a** and **4a** were investigated by determining the nucleus-independent chemical shift (NICS) [42] values and displaying the anisotropy of the induced current density (ACID) (Figs S6–S8) [43]. The results show that **3a** has aromatic character, mainly in the two five-membered rings (Os, C1–C7), providing an extra contribution to its stability. However, as for **4a**, its two five-membered rings (Os, C1–C7) are not aromatic. In addition, the anti-aromatic four-membered ring may decrease its stability. This may cause the difference of stabilities between **3a** and **4a**.

### Theoretical studies on the bonding situation of the Os–C bonds

**Figure 3.**
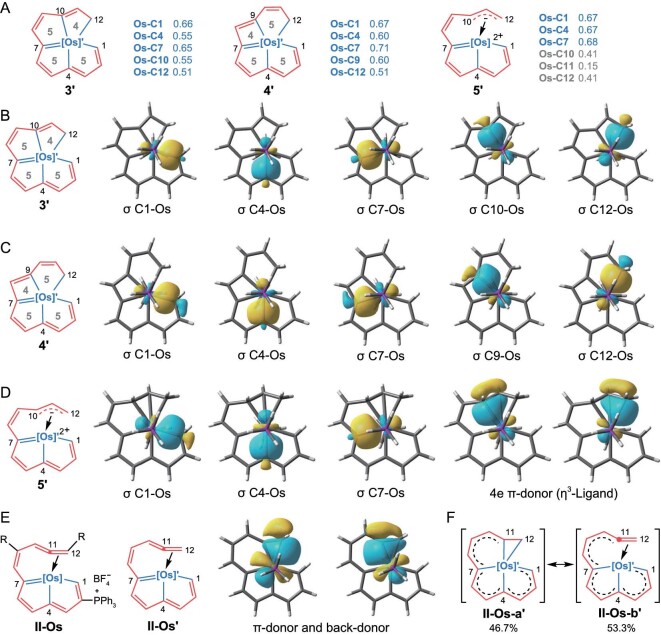
*σ*-Bond analysis of models **3′, 4′, 5′** and **II-Os′**. (A) Wiberg bond index (WBI) of **3′ 4′** and **5′** obtained with NBO 7.0 software. Selected Pipek-Mezey localized molecular orbitals (PM-LMO) of **3′** (B), **4′** (C) and **5′** (D) in *σ*-bond forms; isovalue = 0.05. (E) Structures of **II-Os** and **II-Os′**, and two PM-LMOs of **II-Os′** with a π-ligand form, corresponding to a donor and a back-donor. (F) Ratio of the resonance structural forms of **II-Os-a′** and **II-Os-b′**, and dashed bonds from C1 to C10 in two resonance forms indicate a series of resonance structures they represent. [Os]’  = Os(PH_3_)_2_, [Os]  = Os(PPh_3_)_2_, R = methyl, phenyl or 3-thiophenyl *et al*.

DFT calculations were carried out to reveal the bonding situation of the metal and the carbon atoms. Using the NBO 7.0 software package, the Wiberg bond index (WBI) [44,45] of the M–C bonds was determined in **3′, 4′** and **5′**, three simplified skeletons of **3a, 4a**, and **5a** (Fig. 3A). Relatively large WBI numbers indicate the presence of M–C *σ* bonds. Small WBI numbers, such as those for C10, C11 and C12 in **5′**, denote weak bonds and indicate *η^3^*-ligand coordination to osmium.

To further describe the M–C *σ* bonds, the Pipek-Mezey localized molecular orbitals (PM-LMOs) [46] were determined using the Multiwfn software package [47]. This analysis shows five M–C *σ* bonds on the equatorial plane of **3′** and **4′** (Fig. 3B and 3C), while in **5′**, only three such bonds are seen (Fig. 3D). Two other PM-LMOs on C10–C12 together show a four-electron *η^3^*-ligand coordinated to the osmium center in **5'**.

For comparison, a similar analysis was performed for **II-Os'** as the simplified model for the reported complexes **II-Os** (Fig. 1 and Fig. 3E, left) [35]. Two LMOs of **II-Os'** were identified to engage in a combination of *π* donation and *π* back-donation (Fig. 3E, right). A natural resonance theory (NRT) analysis [48] was performed to determine the proportion of each resonance structure of **II-Os′**. There are several resonance situations of C1 to C10; however, to focus on the part we are interested in, C11, C12 and Os, these resonance structures were combined and described using dashed bonds as shown in Fig. 3F. The results show that the resonance structures with the form of **II-Os-b′** (53.3%) is dominant, relative to ones with the form of **II-Os-a′** (46.7%). These results suggest that the C11–C12 moiety can be viewed as mainly a *π*-ligand, which is further supported by the C10–C11 (1.362(8) Å) and C11–C12 (1.394(8)  Å) bond lengths of one example of **II-Os** (R = Me) in the crystal structure [35]. On the basis of these results, it was therefore concluded that in our previously reported complexes **II-Os**, there are mainly three coplanar Os–C *σ* bonds, and the role of C11 and C12 with Os are primarily *π* interactions, although the *σ* interactions cannot be ignored. Therefore, the skeleton of complexes **II-Os** is totally different from those of **3** and **4**.

**Figure 4.**
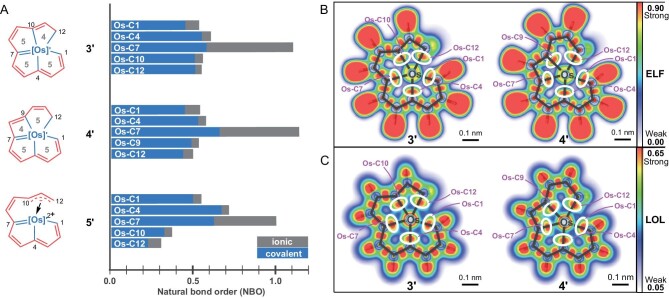
Bond analysis of covalent components and M–C bond visualization. (A) Natural bond order (NBO) analysis of **3′, 4′** and **5′** showing the contributions to bonds. (B) Electron localization function (ELF) graphs of **3′** and **4′**. (C) Localized orbital locator (LOL) graphs of **3′** and **4'**. The strong interactions in circles indicate the bonding force between osmium and each carbon.

Additional DFT computational analyses were performed both with numerical and visual methods to investigate the *σ* character of the M–C bonds in **3′** and **4′**. Usually, bonds can be divided into two main types: ionic and covalent (*σ, π*, etc.). To investigate the covalent and ionic characters of the Os–C bonds in these complexes, the Hirshfeld charges of atoms were calculated first with Multiwfn. The numbers of charges on osmium of **3′** and **4′** are, respectively, 0.007 and 0.002, nearly zero, indicating a non-ionic form. Natural bond order (NBO) analyses were then performed (Fig. 4A) [44] and **5'** was also used as a reference. In **3′** and **4′**, five large Os–C bond orders were observed, indicating strong bonding forces between carbon and osmium. All of the bonds in **3′, 4′** and **5′** except for the Os–C7 bonds had a predominantly covalent character, indicating that the M–C bonds are dominated by a *σ*-covalent rather than ionic component. The large NBO values of Os–C7 in **3′, 4′** and **5′** indicate that the Os–C7 bonds are double bonds, containing not only *σ* character but also *π* character. The half ionic NBO values of Os–C7 might be because its *π* electrons participate in the delocalization of the rings, resulting in less covalent properties. The NBO values of Os–C10 and Os–C12 in skeleton **5′** are lower than those in **3′** because the bonds in the former are *η^3^*-coordination bonds rather than *σ* bonds. This further corroborates the line drawings in Fig. 4A.

Electron localization function (ELF) [49] and localized orbital locator (LOL) [50] analyses of both **3′** and **4′** were also performed, and the results are shown here as visual evidence (Fig. 4B and 4C). The red parts in white cycles revealed the electron localizations between the carbons and osmium, further confirming the existence of five M–C *σ* bonds in each structure. Furthermore, by simply comparing the color gradients in ELF graphs, the electron densities of the M–C *σ* bonds are nearly the same level of those C–C bonds, indicating a possible stability of the M–C bonds. Thus, the results of these DFT calculations are proof of the existence of 5 coplanar M–C *σ* bonds in the [5554] and [5545] structures.

## CONCLUSIONS

To summarize, we have described the synthesis and characterization of two kinds of complexes with five coplanar M–C *σ* bonds. To keep the bonds stable and fix them in a single plane, we developed structures that were expected to have both rigidity and conjugation. As a result, those complexes were found to be exceptionally stable. The existence of the five coplanar M–C *σ* bonds was supported by both experimental and computational data. Note that the M–C bonds are not only *σ* characteristic, but some also have *π* component and give out a large conjugation system. We not only synthesized several new structures in this research, but also expanded the coplanar M–C *σ* bonds to five.

## MATERIALS AND METHODS

Detailed materials and methods are available in the Supplementary data.

## Supplementary Material

nwad325_Supplemental_Files
