# Supplementary material for: Experimental and theoretical evidences for the formation of transition metal complexes with five coplanar metal–carbon σ bonds
Source: Natl Sci Rev. 2023 Dec 20;10(12):nwad325. doi: 10.1093/nsr/nwad325 (PMC10789241; doi:10.1093/nsr/nwad325)
Supplement: nwad325_Supplemental_Files [file nwad325_supplemental_files.zip › Supplementary data-Revised.pdf]

Supplementary Materials

Experimental and theoretical evidences for the formation of transition metal complexes with five coplanar metal–carbon σ bonds

Yuhui Hua[1], Ming Luo[1], Zhengyu Lu[1], Hong Zhang[2], Dafa Chen[1] and Haiping Xia[1,*]

[1] Shenzhen Grubbs Institute and Guangdong Provincial Key Laboratory of Catalysis, Department of Chemistry, Southern University of Science and Technology, Shenzhen 518055, P. R. China.

[2] Department of Chemistry, College of Chemistry and Chemical Engineering, Xiamen University, Xiamen 361005, P. R. China.

[*] Corresponding author. E-mail: xiahp@sustech.edu.cn

Table of Contents

1. Materials and Methods S3

2. Supplementary Figures and Tables to Main Text S4

3. Synthesis Details S15

4. Other Supplementary Figures and Tables S23

5. Computational Cartesian Coordinates S60

6. References S64

1. Materials and Methods

Materials

Complexes **1**, **1c**, **1d**, phenyl ethynyl ether, and 2-ethyl-1-butene-3-yne were synthesized with the method reported.[1-4] Reagents and solvents were used as received from commercial sources without further purification. Column chromatography was performed on alumina gel (200–300 mesh) or silica gel (200–300 mesh) in air.

Methods

All syntheses were performed under air unless otherwise stated. Each synthesis was further followed with a simple flash column with dichloromethane/methanol = 10:1 unless otherwise stated. Nuclear magnetic resonance (NMR) spectroscopic experiments were performed on a Bruker AV-600 spectrometer (600 MHz) or Bruker AV-400 spectrometer (400 MHz) at room temperature. The ^{1}H and ^{13}C NMR chemical shifts (δ) are relative to tetramethylsilane, and the ^{31}P NMR chemical shifts are relative to 85% H_3PO_4. The absolute values of the coupling constants are given in hertz (Hz). Multiplicities are abbreviated as singlet (s), doublet (d), triplet (t), multiplet (m), quartet (q) and broad (br). High-resolution mass spectra (HRMS) experiments were recorded on a Bruker En Apex Ultra 7.0T Fourier Transform Mass Spectrometer. The theoretical molecular ion peak was calculated by Compass Isotope Pattern software supplied by Bruker Co.

All the calculations of structural optimizations were performed at M06-L/Def2-SVP of density functional theory (DFT).[5,6] Frequency calculations were performed at the same level of theory as for geometry optimization to characterize the stationary points as either minima (no imaginary frequencies). All the calculations were performed with the Gaussian 16 software package, Revision A.03.[7] Nucleus-independent chemical shifts (NICS) values were calculated at the same level.[8] The anisotropy of the induced current density (ACID) calculations was carried out with the ACID program.[9] The analysis of the Wiberg bond index (WBI), natural resonance theory (NRT), and natural bond order (NBO) was performed with NBO 7.0 software package.[10] The non-covalent interaction (NCI) analysis was performed directly using the structures from X-ray crystal data. All the isosurfaces were visualized by Multiwfn 3.8.[11]

Single crystals suitable for X-ray diffraction were from a mixture of dichloromethane and chloroform solution with layered hexane. The diffraction data of **2**, **3a**, **4a** and **5a** were recorded on Agilent SuperNova X-Ray single crystal diffractometer with mirror-monochromated Cu Kα radiation (λ = 1.54184 Å), and the diffraction data of **3b** and **4b** were recorded on Bruker APEX-II CCD with graphite-monochromated Cu Kα radiation (λ = 1.54178 Å). These structures were solved with Olex2[12] by ShelXT[13] structure solution program by intrinsic phasing method, and all of them were refined with the ShelXL[14] refinement package using least-squares minimization. All non-hydrogen atoms were refined anisotropically unless otherwise stated. The hydrogen atoms were placed at their idealized positions and assumed the riding model unless otherwise stated. The diffuse electron densities resulting from the residual solvent molecules in complexes **2** and **4b** were removed from the data set by using the SQUEEZE routine of PLATON. X-ray crystal structure information is available at the Cambridge Crystallographic Data Centre (CCDC) under deposition numbers CCDC 2165154 (**2**), 2165155 (**3a**), 2165156 (**3b**), 2165157 (**4a**), 2165158 (**4b**), 2165159 (**5a**). For further details on the crystal data, data collection, and refinements, see Table S1 and S2.

2. Supplementary Figures and Tables to Main Text

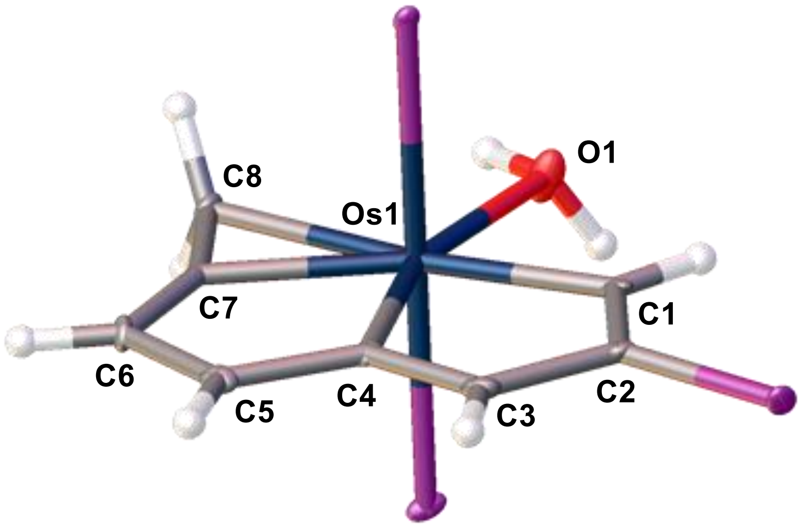

Figure S1. X-ray molecular structure for the cation of complex **2** drawn with 50% probability level. The phenyl groups in PPh_3 are omitted for clarity. Selected bond lengths [Å] and angles [°]: Os1–O1 2.249(3), Os1–C1 2.037(4), Os1–C4 2.072(4), Os1–C7 1.999(4), Os1–C8 2.267(4), C1–C2 1.384(6), C2–C3 1.417(6), C3–C4 1.378(6), C4–C5 1.404(6), C5–C6 1.390(6), C6–C7 1.377(6) , C7–C8 1.389(7); Os1–C1–C2 118.6(3), C1–C2–C3 114.1(4), C2–C3–C4 113.3(4), C3–C4–Os1 118.0(3), C1–Os1–C4 75.94(16), Os1–C4–C5 118.8(3), C4–C5–C6 113.3(4), C5–C6–C7 110.3(4), C6–C7–Os1 124.5(3), C7–Os1–C4 73.04(17), Os1–C7–C8 81.9(3), C7–C8–Os1 60.8(2).

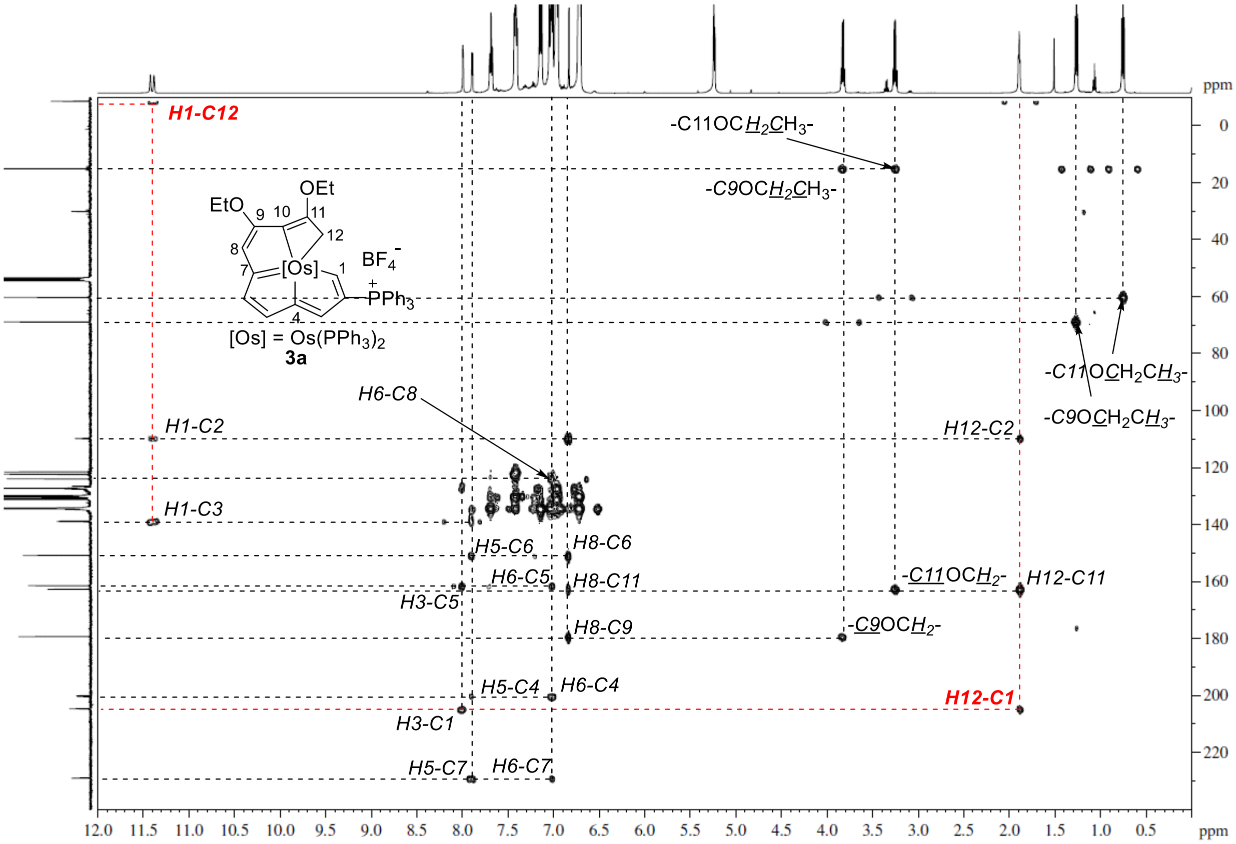

Figure S2. Two-dimensional ^{1}H-^{13}C-HMBC spectrum of **3a** in CD_2Cl_2. The strong correlation between H1 and C12, H12 and C1 are shown in red marks.

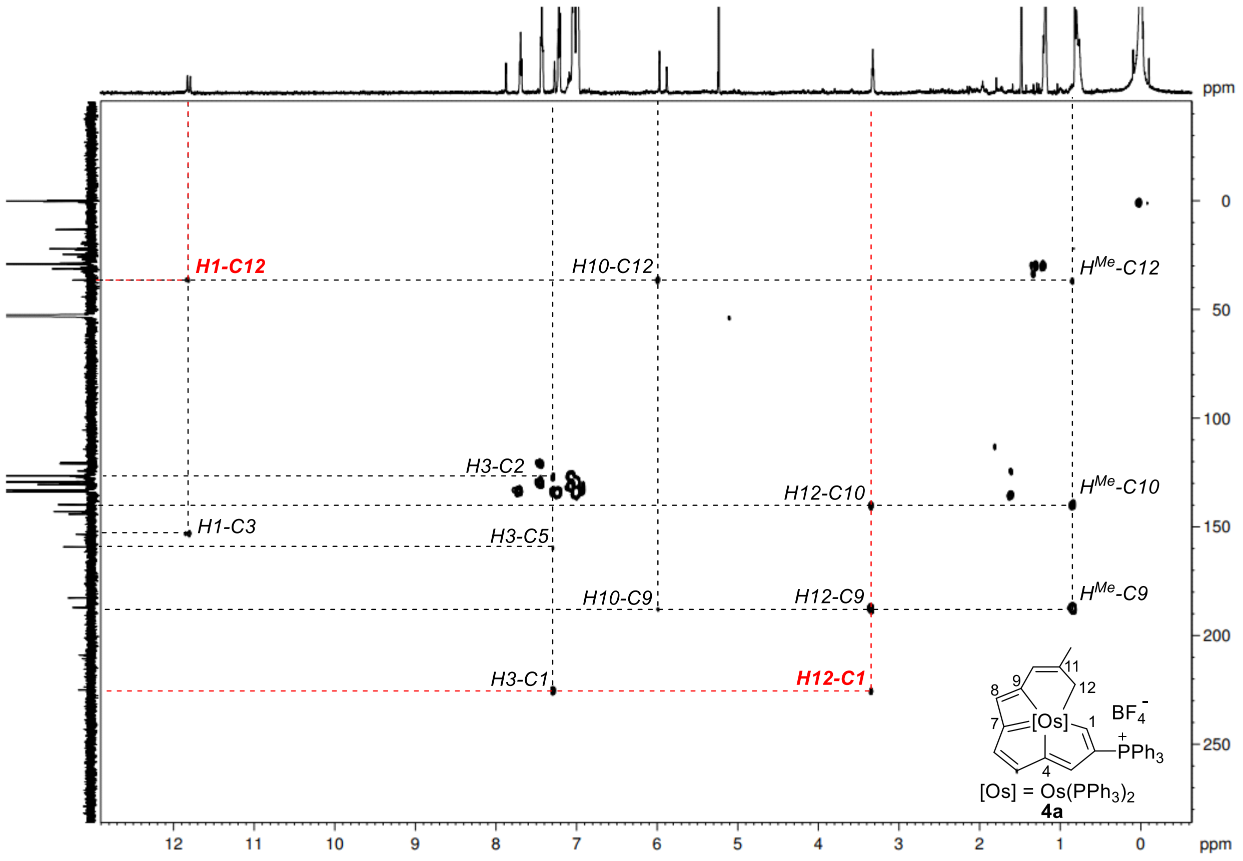

Figure S3. Two-dimensional ^1H-^{13}C-HMBC spectrum of **4a** in CD_2Cl_2. The strong correlation between H1 and C12, H12 and C1 are shown in red marks.

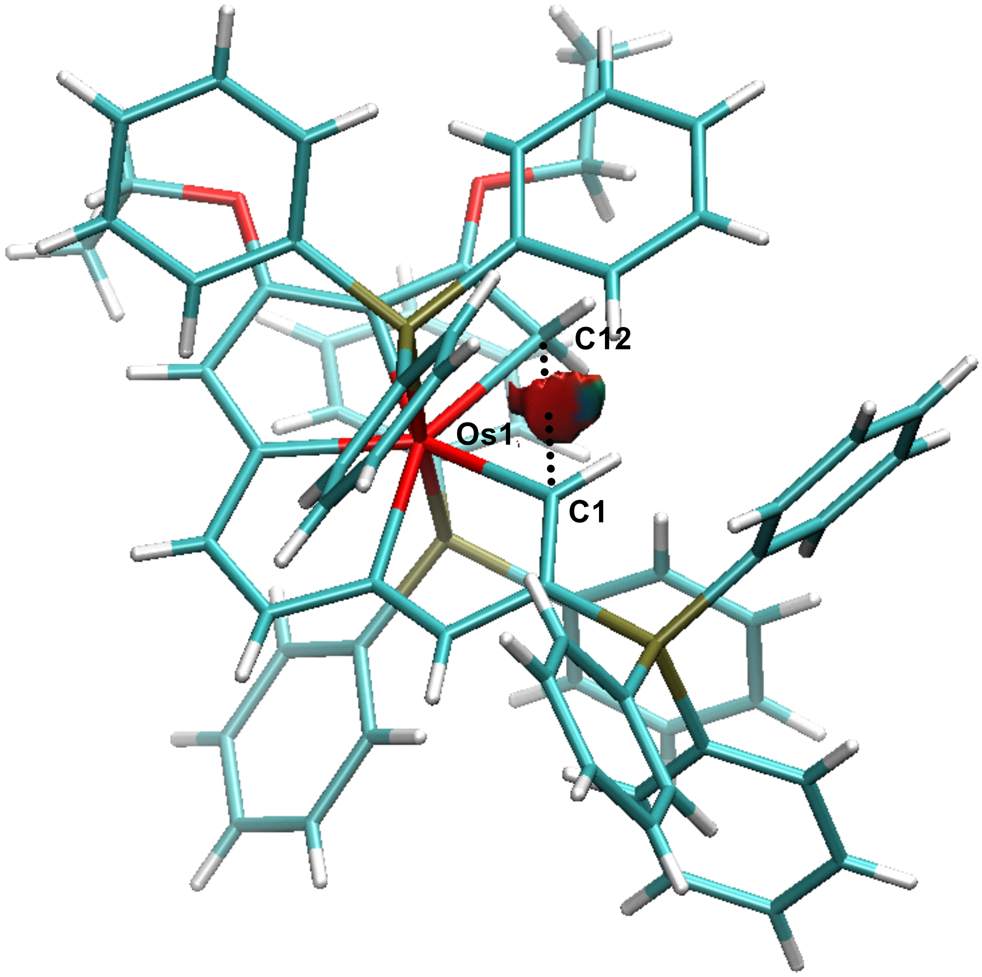

Figure S4. NCI analysis of **3⁺ cation**. The red surface indicates the repulsion between C12 and C1 sites.

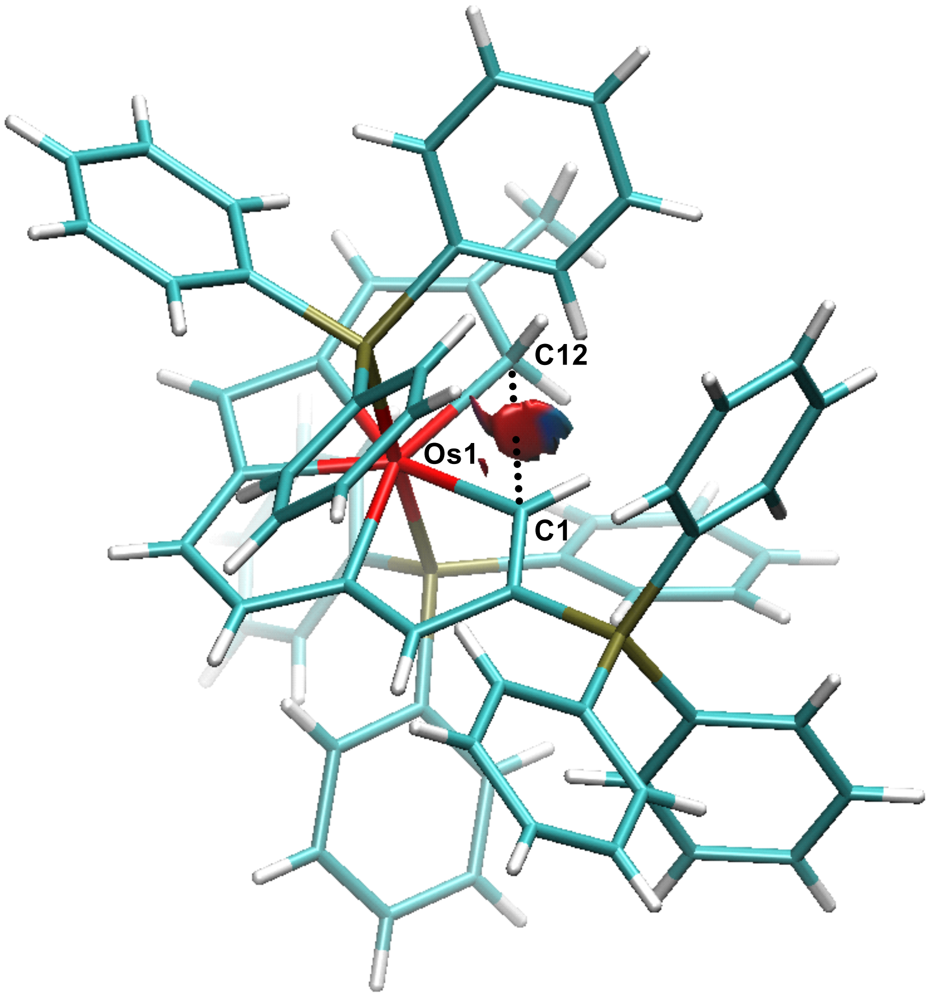

Figure S5. NCI analysis of **4^+ cation**. The red surface indicates the repulsion between C12 and C1 sites.

Table S1. Stability studies of **3a** and **4a** (solid, in air, min = minute, h = hour, d = day, m = month).

Percentages mean the remaining of materials (tested with ^{31}P-NMR).

**Compound**	**T/ºC**	**1 h**	**1 d**	**3 d**	**7 d**	**1 m**	**3 m**	**6 m**
**3a**	r.t.	100%	100%	100%	100%	100%	> 99%	> 99%
**4a**	r.t.	100%	100%	100%	> 99%	> 95%	~ 90%	~80%
**Compound**	**T/ºC**	**30 min**	**1 h**	**3 h**	**12 h**	**1 d**	**2 d**	**5 d**
**3a**	50	100%	100%	100%	100%	100%	100%	> 99%
**4a**	50	> 99%	> 99%	> 95%	~ 90%	~ 80%	-	-
**3a**	80	100%	100%	100%	100%	100%	> 99%	> 95%
**3a**	100	100%	100%	100%	> 99%	> 95%	~ 95%	~ 90%

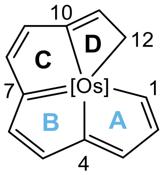

3'

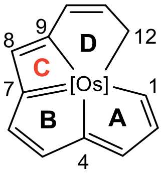

4'

Struct.	$NICS(1)_{zz}$			
	Ring A	Ring B	Ring C	Ring D
3'	-17.2	-15.1	-2.6	+4.0
4'	+7.2	-4.0	+22.6	-5.4

[Os] = $Os(PH_3)_2$

Figure S6. Nucleus-independent chemical shift (NICS) values [$NICS(1)_{zz}$] of skeleton **3’** and **4’**. Big positive numbers indicate anti-aromaticity and negative ones indicate aromaticity. The numbers near zero indicate non-aromaticity.

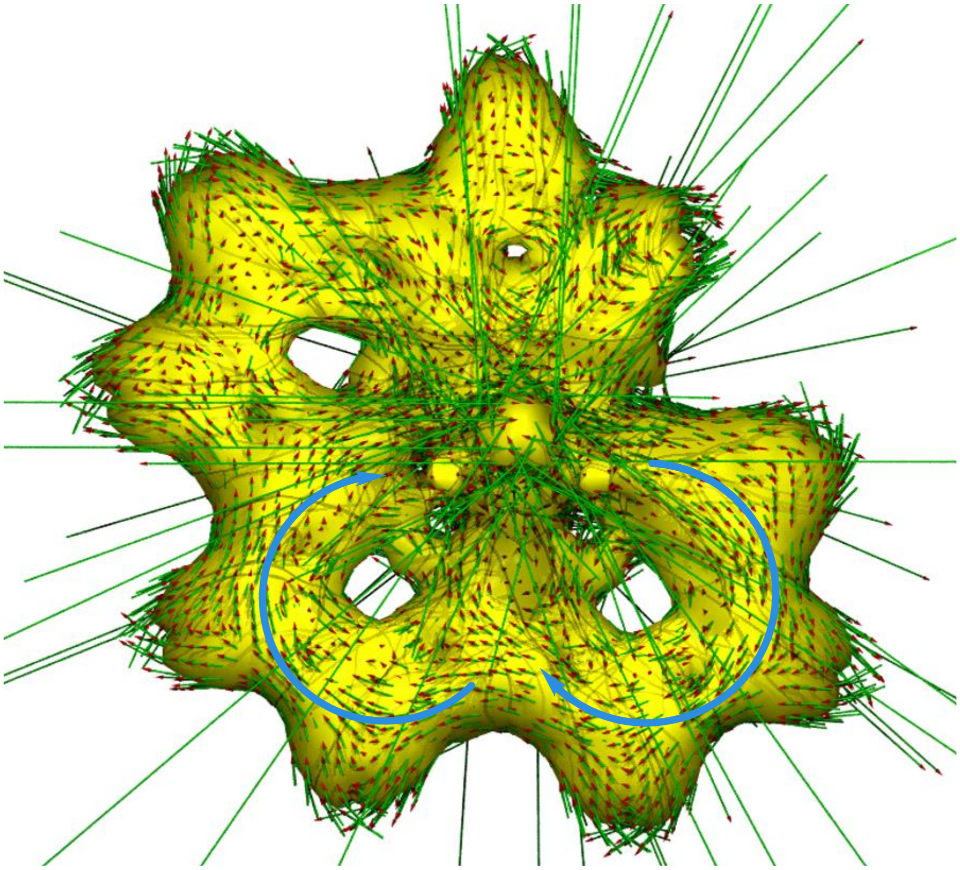

Figure S7. Anisotropy of the induced current density (ACID) graph of **3’**. The clockwise current indicates aromaticity. Isovalue = 0.030. (**3’** is a simplified skeleton of **3a**, see Figure S6 above.)

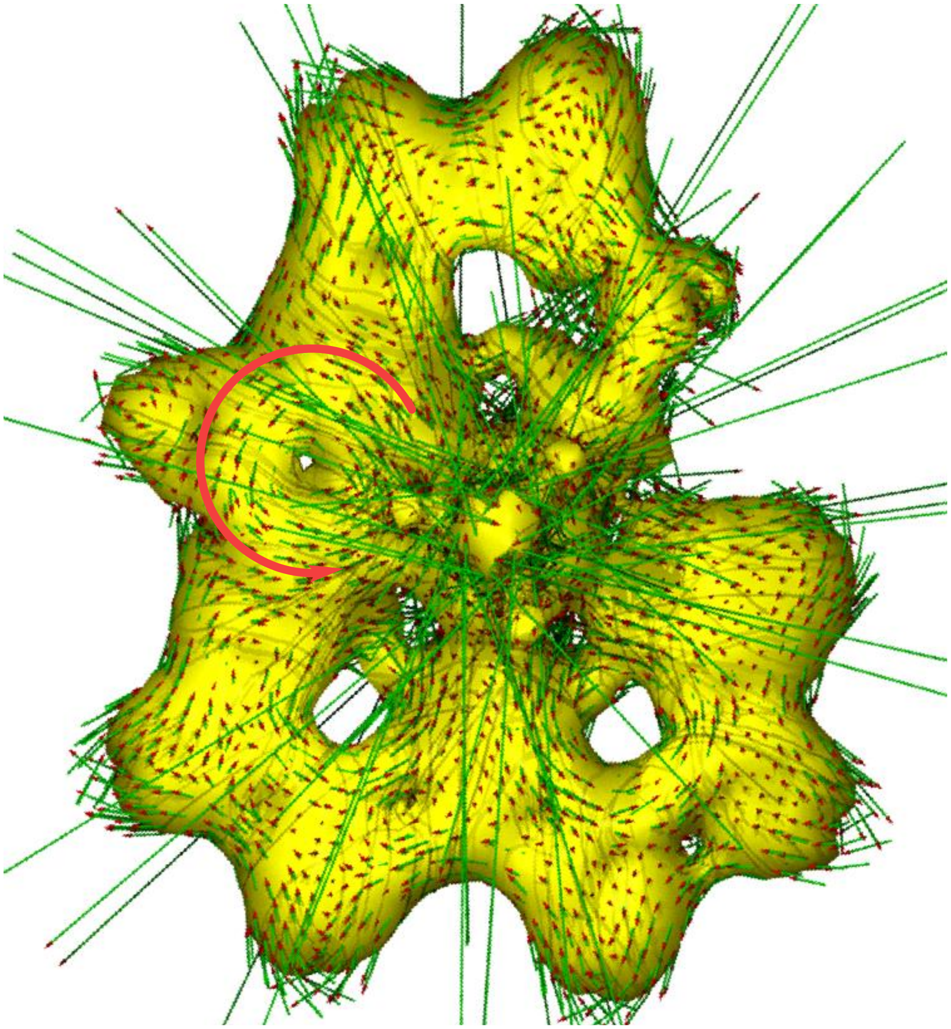

Figure S8. Anisotropy of the induced current density (ACID) graph of **4'**. The anti-clockwise current indicates anti-aromaticity. Isovalue = 0.030. (**4'** is a simplified skeleton of **4a**, see Figure S6 above.)

Table S2. Atomic contributions to LMOs of C-M bonds in **3’** and **4’**. A big C/M contribution indicates a polarized bond that is much like a pair of carbon anion and metal cation.

**Name**	**3’**		**4’**	
**Structure**	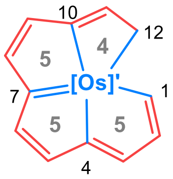<br><br>**3'**		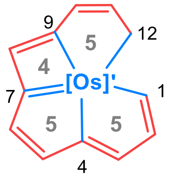<br><br>**4'**	
**Atomic Contributions to LMO (%)**	**C1/Os**	54.7/31.8	**C1/Os**	55.7/30.5
	**C4/Os**	51.6/31.1	**C4/Os**	51.2/32.3
	**C7/Os**	51.9/32.9	**C7/Os**	47.5/36.0
	**C10/Os**	46.3/36.3	**C9/Os**	47.8/35.0
	**C12/Os**	45.6/36.5	**C12/Os**	50.4/33.0
**Ratios of Contributions**	**C1/Os**	1.72	**C1/Os**	1.83
	**C4/Os**	1.66	**C4/Os**	1.59
	**C7/Os**	1.58	**C7/Os**	1.32
	**C10/Os**	1.28	**C9/Os**	1.37
	**C12/Os**	1.25	**C12/Os**	1.53
**Average Ratio**	**C/Os**	1.50	**C/Os**	1.53
**Result**	**Covalent bonds**		**Covalent bonds**	

Table S3. Electron density and energy density at the bond-critical point (bcp) analyses for skeletons **3’** and **4’**. A big number of ρ_{bcp} or a minus number of $E(r)_{bcp}$ usually indicate the covalent bond.

**Name**	**3’**	**4’**
**Structure**	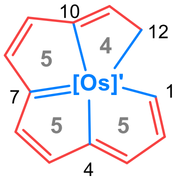 **3'**	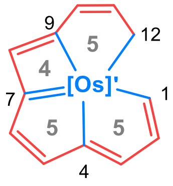 **4'**
**ρ_{bcp}** Average Electron Density of $[C\text{-}M]_{bcp}$	0.12	0.12
**$E(r)_{bcp}$** Average Energy Density of $[C\text{-}M]_{bcp}$	-0.06	-0.06
**Result**	Covalent	Covalent

3. Synthesis Details

3.1 General syntheses scopes of [5554] and [5545]

General synthesis scope of [5554] complexes **3.**

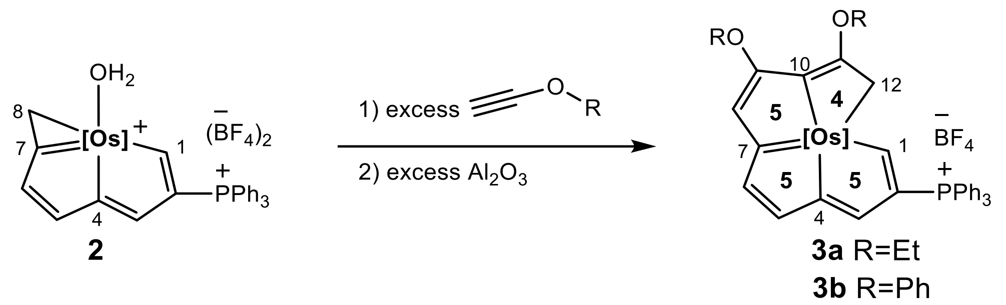

General synthesis scope of [5545] complexes **4.**

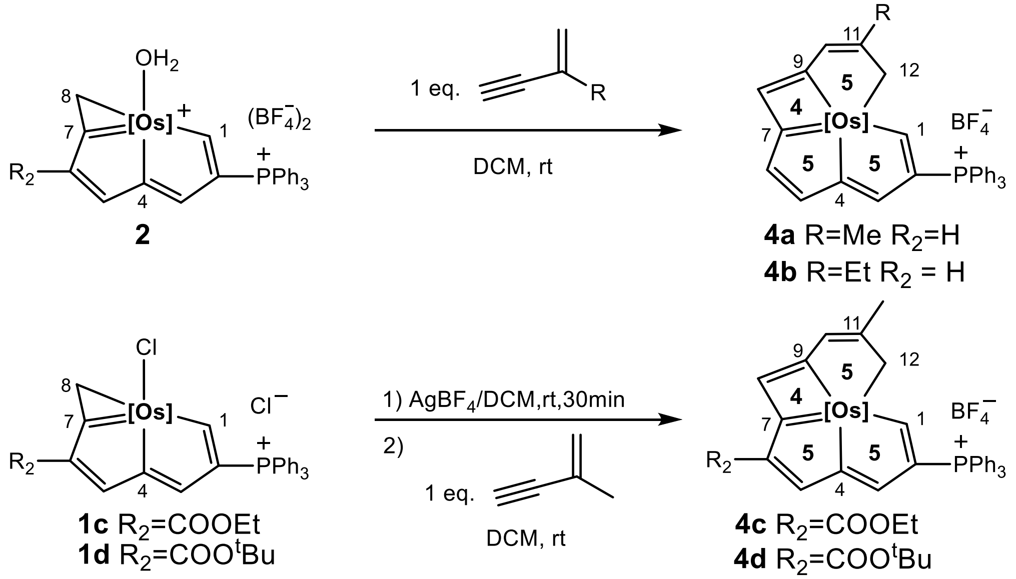

3.2 Syntheses of complexes 2 to 5a.

Synthesis of complex **2**.

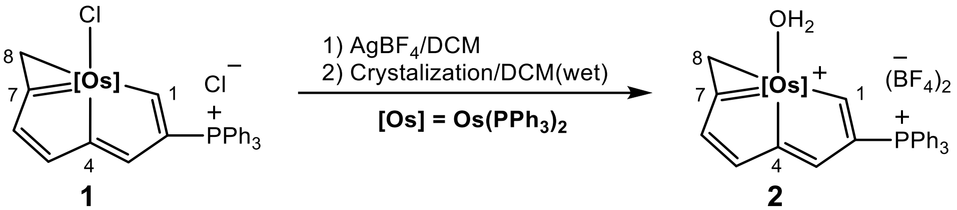

AgBF$_4$ (584 mg, 3.00 mmol) was added into a solution of complex **1** (1.15 g, 1.00 mmol) in CH_2Cl_2 (12 mL) in air. The reaction mixture was stirred at room temperature for 30 mins to yield a brown solution mixed with Ag-salt (AgCl) solid. The filtration was then carried out with diatomite to remove AgCl, and a limpid brown solution was obtained. Extra CH_2Cl_2 was then added to this solution until the volume reached approximately 20 mL. After about 1 day of standing in air, many crystals precipitated from the solution. The dark orange crystals of 8C-carbolong complex **2** (571 mg, 45% yield) were collected by filtration and then washed by DCM (10 mL). ^{1}H NMR (400.0 MHz, CD_2Cl_2 with 10% CD_3OD, ppm): δ = 14.10 (d, 1H, H1, $J_{H\text{-}P}$ = 16.5 Hz), 9.14 (s, 1H, H5), 8.70 (s, 1H, H3), 6.81(s, in other peaks confirmed by HSQC, H6), 6.75 (br, in other peaks confirmed by HMBC), 3.06 (br, 2H, H8), other peaks at 6.00-8.00 ppm are contributed to protons of phenyl groups. ^{31}P NMR (161.9 MHz, CD_2Cl_2 with 10% CD_3OD, ppm): δ = 12.24 (s, C2*P*Ph$_3$), -4.96 (s, Os*P*Ph$_3$). ^{13}C NMR (100.1 MHz, CD_2Cl_2 with 10% CD_3OD, plus ^{13}C DEPT-135, ^{1}H-^{13}C HSQC and ^{1}H-^{13}C HMBC, ppm): δ = 238.9 (br, C7), 237.6 (m, C1), 189.5 (d, C4, $J_{P\text{-}C}$ = 23.3 Hz), 167.3 (s, C5), 151.8 (d, C3, $J_{C\text{-}P}$ = 18.3 Hz), 144.3 (s, C6), 127.7 (d, C2, $J_{C\text{-}P}$ = 52.4 Hz) , 27.7 (s, C8), other peaks at 120.0-140.0 ppm are contributed to carbons of phenyl groups. Anal. Calcd (%) for $C_{62}H_{53}B_2F_8OOsP_3$: C, 58.60; H, 4.20; Found: C, 58.39; H, 3.96.

Synthesis of complexes **3a** and **5a**.

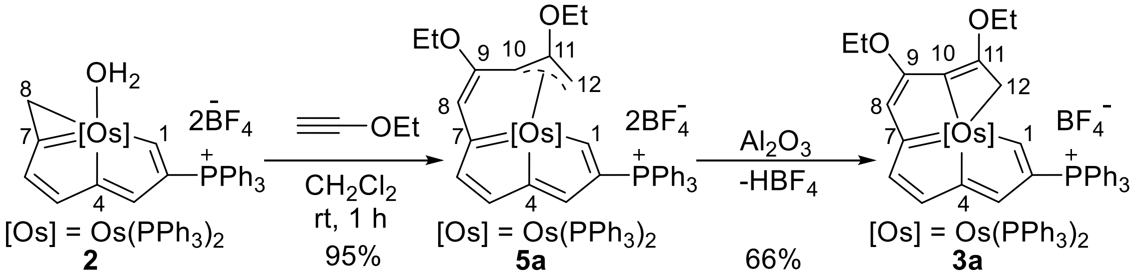

About 5 eq. of ethyl ethynyl ether (55 wt% in hexane, about 0.8 mL) was added into a solution of complex **2** (1.27 g, 1.00 mmol) in CH_2Cl_2 (12 mL) in air. The reaction mixture was stirred at room temperature for 1 hour to yield a purple solution. Then the solution was reduced to about 3 mL under vacuum, in which ethyl ether (20 mL) was next added to give some precipitate. The precipitate was collected by filtration and washed with ethyl ether (2 × 20 mL), affording **5a** (1.32 g, 95% yield) as a purple solid.

Excess Al_2O_3 was added into a solution of complex **5a** (1.32 g) in CH_2Cl_2 (12 mL), and the resulted solution was stirred for 30 mins to yield a green mixture. The solvent was dried under vacuum, and the residue was purified by column chromatography on alumina gel (eluent: dichloromethane/methanol = 15:1 to 5:1), affording **3a** (860 mg, 66% yield) as a green solid.

For **5a:** ^{1}H NMR (600.1 MHz, CD_2Cl_2, ppm): δ = 13.42 (d, 1H, H1, J_{P-H} = 18.1 Hz), 8.24 (d, 1H, H5, J_{H-H} = 4.2 Hz), 8.00 (weak d or br, 1H, H3), 7.28 (m, 1H, H6), 6.61 (s, 1H, H8), 4.97 (d, 1H, H12b, J_{H-H} = 5.0 Hz), 4.55 (dt, 1H, H12a, J_{H-H} = 5.7 Hz, J_{P-H} = 8.5 Hz), 3.96 (m, 2H, $OC\mathit{H}_2CH_3$), 3.79 (m, 1H, $OC\mathit{H}_2CH_3$), 2.77 (m, 1H, H10), 2.76 (m, 1H, $OC\mathit{H}_2CH_3$), 1.35 (m, 3H, $OCH_2C\mathit{H}_3$), 0.68 (m, 3H, $OCH_2C\mathit{H}_3$), other peaks at 6.00-8.00 ppm are contributed to protons of phenyl groups. ^{31}P NMR (242.9 MHz, CD_2Cl_2, ppm): δ = 10.45 (s, C2*P*Ph_3), -9.08 and -18.64 (both d, Os*P*Ph_3, J_{P-P} = 261.4 Hz). ^{13}C NMR (150.9 MHz, CD_2Cl_2, plus ^{13}C DEPT-135, ^{1}H-^{13}C HSQC and ^{1}H-^{13}C HMBC, ppm): δ = 230.4 (dd, J_{C-P} = 8.9 Hz, J_{C-P} = 5.7 Hz, C7), 196.5 (d, C1, J_{P-C} = 18.1 Hz), 194.2 (m, C4), 177.0 (s, C9), 166.6 (s, C5), 162.3 (d, J_{C-P} = 3.0 Hz, C11), 152.5 (s, C6), 150.9 (d, J_{C-P} = 22.6 Hz, C3), 133.2 (in other peaks, confirmed by HMBC, C2), 128.8 (s, C8), 70.3 (s, $O\mathit{C}H_2CH_3$), 70.0 (s, $O\mathit{C}H_2CH_3$), 63.7 (s, C10), 41.3 (s, C12), 13.6 (s, $OCH_2\mathit{C}H_3$), 13.5 (s, $OCH_2\mathit{C}H_3$), other peaks at 110.0-140.0 ppm are contributed to carbons of phenyl groups. Anal. Calcd (%) for $C_{70}H_{62}B_2F_8O_2OsP_3$: C, 60.31; H, 4.63; Found: C, 60.42; H, 4.507.

For **3a:** ^{1}H NMR (400.0 MHz, CD_2Cl_2, ppm): δ = 11.40 (dd, 1H, H1, J_{P-H} = 16.2 Hz, J_{P-H} = 1.56 Hz), 7.99 (d, 1H, H3, J_{P-H} = 2.0 Hz), 7.89 (d, 1H, H5, J_{H-H} = 3.3 Hz), 7.02 (in other peaks, confirmed by HSQC, H6), 6.83 (s, 1H, H8), 3.83 (q, 2H, $OC\mathit{H}_2CH_3$, J_{H-H} = 6.4 Hz), 3.25 (q, 2H, $OC\mathit{H}_2CH_3$, J_{H-H} = 6.4 Hz), 1.89 (t, 2H, H12, J_{P-H} = 3.2 Hz), 1.26 (t, 3H, $OCH_2C\mathit{H}_3$, J_{H-H} = 6.4 Hz), 0.75 (t, 3H, $OCH_2C\mathit{H}_3$, J_{H-H} = 6.4 Hz), other peaks at 6.00-8.00 ppm are contributed to protons of phenyl groups. ^{31}P NMR (162.0 MHz, CD_2Cl_2, ppm): δ = 9.41 (s, C2*P*Ph_3), -9.05 (s, Os*P*Ph_3). ^{13}C NMR (100.1 MHz, CD_2Cl_2, plus ^{13}C DEPT-135, ^{1}H-^{13}C HSQC and ^{1}H-^{13}C HMBC, ppm): δ = 229.0 (t, J_{C-P} = 6.2 Hz, C7), 204.7 (dt, J_{C-P} = 11.0 Hz, J_{C-P} = 4.3 Hz, C4), 200.2 (dt, J_{C-P} = 26.8 Hz, J_{C-P} = 4.4 Hz, C1), 179.3 (s, C9), 162.7 (s, C11), 161.5 (s, C5), 150.8 (s, C6), 138.8 (d, J_{C-P} = 24.2 Hz, C3), 131.0 (t, J_{C-P} = 23.6 Hz, C10), 123.9 (s, C8), 110.0 (t, J_{C-P} = 73.1 Hz, C2), 68.8 (s, $O\mathit{C}H_2CH_3$), 60.5 (s, $O\mathit{C}H_2CH_3$), 15.1 (s, $OCH_2\mathit{C}H_3$), 15.0 (s, $OCH_2\mathit{C}H_3$), -8.7 (t, J_{C-P} = 4.9 Hz, C12), other peaks at 120.0-140.0 ppm are contributed to carbons of phenyl groups. Anal. Calcd (%) for $C_{73}H_{68}BF_4O_4OsP_3$: C, 64.42; H, 4.79; Found: C, 64.72; H, 4.807.

Synthesis of complex **3b**.

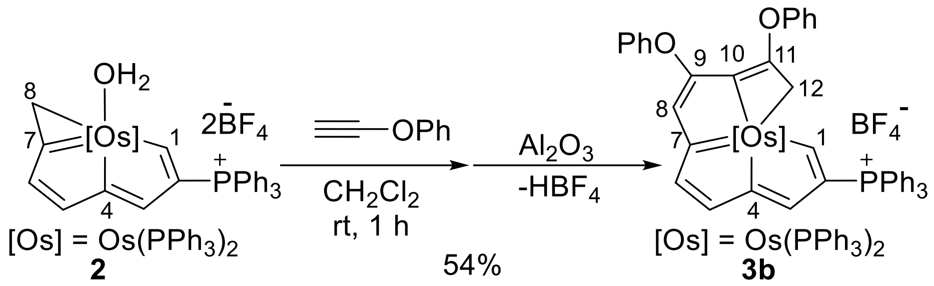

About 4 eq. of phenyl ethynyl ether (0.3 mL) was added into a solution of complex **2** (761 mg, 0.60 mmol) in CH_2Cl_2 (12 mL) in air. The reaction mixture was stirred at room temperature for 1 hour to yield a purple solution. Then the solution was reduced to about 3 mL under vacuum, in which ethyl ether (20 mL) was next added to give some precipitate. The precipitate was collected by filtration and washed with ethyl ether (2 × 20 mL), which then redissolved in CH_2Cl_2 (12 mL). Excess Al_2O_3 was subsequently added, and the resulted solution was stirred for 30 mins to yield a gray green mixture. The solvent was dried under vacuum, and the residue was purified by column chromatography on alumina gel (eluent: dichloromethane/methanol = 10:1), affording **3b** (454 mg, 66% yield) as a grey green solid.

^{1}H NMR (600.1 MHz, CD_2Cl_2, ppm): δ = 11.77 (d, 1H, H1, $J_{P\text{-}H}$ = 19.7 Hz), 8.10 (d, 1H, H5, $J_{H\text{-}H}$ = 7.9 Hz), 8.10 (d, 1H, H3, $J_{H\text{-}H}$ = 6.9 Hz), 6.95 (in peaks, confirmed by HSQC, 1H, H6), 6.68 (s, 1H, H8), 2.16 (t, 2H, H12, $J_{P\text{-}H}$ = 4.1 Hz), other peaks at 6.00-8.00 ppm are contributed to protons of phenyl groups. ^{31}P NMR (242.9 MHz, CD_2Cl_2, ppm): δ = 9.89 (s, C2*P*Ph_3), -10.65 (s, Os*P*Ph_3). ^{13}C NMR (150.9 MHz, CD_2Cl_2, plus ^{1}H-^{13}C HSQC and ^{1}H-^{13}C HMBC): δ = 230.6 (t, C7, $J_{C\text{-}P}$ = 5.9 Hz), 205.1 (td, C1, $J_{P\text{-}C}$ = 10.7 Hz, $J_{P\text{-}C}$ = 3.9 Hz), 200.0 (dt, C4, $J_{P\text{-}C}$ = 26.7 Hz, $J_{P\text{-}C}$ = 4.5 Hz), 174.6 (s, C9), 162.0 (s, C5), 153.2 (s, C11,), 151.4 (s, C6), 140.9 (d, $J_{C\text{-}P}$ = 23.4 Hz, C3), 127.6 (in peaks, confirmed by HMBC, C2), 125.8 (s, C8), 112.0 (t, C10, $J_{C\text{-}P}$ = 9.4 Hz), -8.3 (t, C12, $J_{C\text{-}P}$ = 5.4 Hz), other peaks at 110.0-140.0 ppm are contributed to carbons of phenyl groups. Anal. Calcd (%) for $C_{78}H_{62}BF_4O_2OsP_3$: C, 66.86; H, 4.46; Found: C, 66.77; H, 4.353.

Synthesis of complex **4a**.

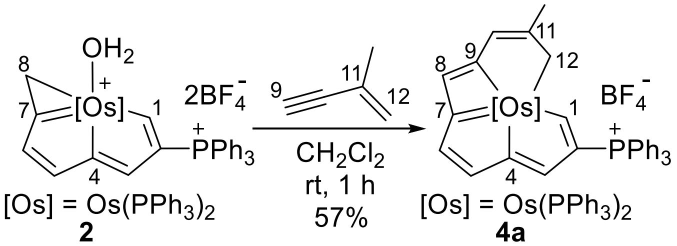

About 1.05 eq. of 2-methyl-1-butene-3-yne (about 45 μL) was added dropwise to a solution of complex **2** (645 mg, 0.51 mmol) in a mixed solvent (CH_2Cl_2/CH_3OH, v:v = 7:1, 8 mL) in argon (Ar) atmosphere. The reaction mixture was stirred at room temperature for 1 hour to yield a greenish brown solution. Then the solution was reduced to about 3 mL under vacuum, in which ethyl ether (20 mL) was next added to give some precipitate. The precipitate was collected by filtration and washed with ethyl ether (2 × 20 mL), affording **4a** (355 mg, 57% yield) as a brown solid.

^{1}H NMR (600.1 MHz, CD_2Cl_2, ppm): δ = 11.81 (d, 1H, H1, $J_{P\text{-}H}$ = 22.4 Hz), 7.87 (d, 1H, H5, $J_{H\text{-}H}$ = 3.5 Hz), 7.28 (br, 1H, H3), 7.02 (in peaks, confirmed by HSQC, 1H, H8), 5.97 (s, 1H, H10), 5.88 (d, 1H, H6, $J_{H\text{-}H}$ = 2.6 Hz), 3.33 (t, 1H, H12, $J_{P\text{-}H}$ = 5.9 Hz), 0.82 (s, 3H, Me), other peaks at 6.00-8.00 ppm are contributed to protons of phenyl groups. ^{31}P NMR (242.9 MHz, CD_2Cl_2, ppm): δ = 8.60 (s, C2*P*Ph$_3$), -1.42 (both d, Os*P*Ph$_3$, $J_{P\text{-}P}$ = 261.4 Hz). ^{13}C NMR (150.9 MHz, CD_2Cl_2, plus ^{1}H-^{13}C HSQC and ^{1}H-^{13}C HMBC, ppm): δ = 224.8 (m, C1), 208.8 (m, C4), 186.9 (s, C9), 182.4 (m, C7), 159.0 (s, C5), 153.1 (d, $J_{C\text{-}P}$ = 25.3 Hz, C3), 143.9 (s, C8), 142.8 (s, C6), 139.6 (s, C10), 131.1 (s, C11), 126.5 (m, C2), 36.3 (m, C12), 24.5 (s, Me), other peaks at 110.0-140.0 ppm are contributed to carbons of phenyl groups. Anal. Calcd (%) for $C_{67}H_{56}BF_4OsP_3$: C, 65.37; H, 4.59; Found: C, 65.20; H, 4.880.

Synthesis of complex **4b**.

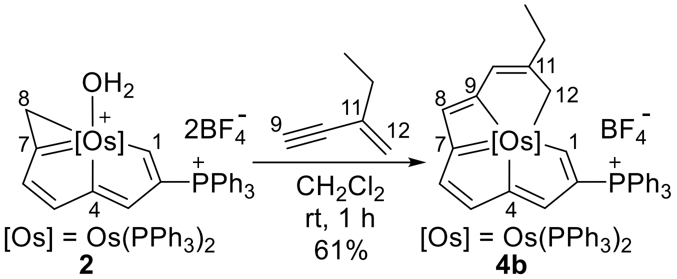

About 1.05 eq. of 2-ethyl-1-butene-3-yne (about 59 μL) was added dropwise to a solution of complex **2** (760 mg, 0.60 mmol) in a mixed solvent (CH_2Cl_2/CH_3OH, v:v = 7:1, 8 mL) in argon (Ar) atmosphere. The reaction mixture was stirred at room temperature for 1 hour to yield a greenish brown solution. Then the solution was reduced to about 3 mL under vacuum, in which ethyl ether (20 mL) was next added to give some precipitate. The precipitate was collected by filtration and washed with ethyl ether (2 × 20 mL), affording **4b** (451 mg, 61% yield) as a brown solid.

^{1}H NMR (600.1 MHz, CD_2Cl_2, ppm): δ = 11.84 (d, 1H, H1, $J_{P\text{-}H}$ = 20.9 Hz), 7.89 (d, 1H, H5, $J_{H\text{-}H}$ = 3.5 Hz), 7.29 (br, 1H, H3), 7.01 (br, 1H, H6), 6.02 (s, 1H, H10), 5.86 (s, 1H, H8), 3.37 (t, 2H, H12, $J_{P\text{-}H}$ = 6.7 Hz), 1.10 (q, 2H, CH_2CH$_3$, $J_{H\text{-}H}$ = 7.4 Hz), 0.47 (t, 3H, CH$_2$CH_3, $J_{H\text{-}H}$ = 7.4 Hz), other peaks at 6.00-8.00 ppm are contributed to protons of phenyl groups. ^{31}P NMR (242.9 MHz, CD_2Cl_2, ppm): δ = 8.58 (s, C2*P*Ph$_3$), -1.58 (s, Os*P*Ph$_3$). ^{13}C NMR (150.9 MHz, CD_2Cl_2, ppm): δ = 225.8 (m, C1), 203.4 (m, C4), 193.4 (s, C9), 183.3 (m, C7), 159.8 (s, C5), 154.1 (d, C3, $J_{P\text{-}C}$=25.5 Hz), 145.1 (s, C6), 145.1 (s, C11), 143.6 (s, C8), 138.6 (s, C10), 127.8 (m, C2), 34.9 (m, C12), 33.1 (s, *C*H$_2$CH$_3$), 12.0 (s, CH$_2$*C*H$_3$), other peaks at 110.0-140.0 ppm are contributed to carbons of phenyl groups. Anal. Calcd (%) for $C_{68}H_{58}BF_4OsP_3$: C, 65.59; H, 4.70; Found: C, 65.80; H, 4.495.

Synthesis of complex **4c**.

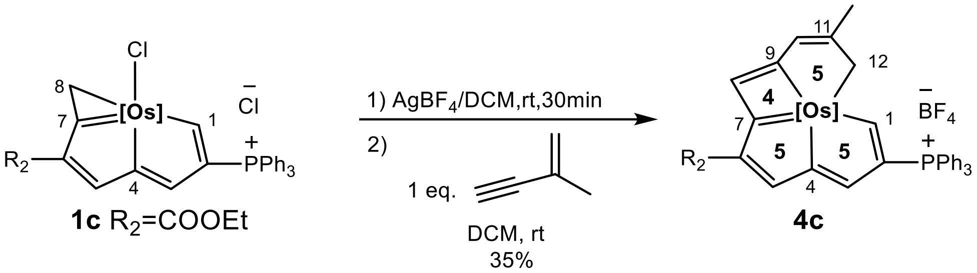

$AgBF_4$ (293 mg, 1.5 mmol) was added into a solution of complex **1c** (611 mg, 0.5 mmol) in CH_2Cl_2 (10 mL) in argon (Ar) atmosphere. The reaction mixture was stirred at room temperature for 30 mins to yield a brown solution mixed with Ag-salt (AgCl) solid. The filtration was then carried out with diatomite to remove AgCl, and a limpid brown solution was obtained, which was added with some additional CH_2Cl_2 (10 mL). Subsequently, about 1.05 eq. of 2-methyl-1-butene-3-yne (about 10 μL) was added dropwise into the solution in argon (Ar) atmosphere. After stirring at room temperature for 1 hour, a dark brown solution was generated. Then the solution was reduced to about 3 mL under vacuum, in which ethyl ether (20 mL) was next added to give some precipitate. The precipitate was collected by filtration and washed with ethyl ether (2 × 20 mL), affording **4c** (229 mg, 35% yield) as a brown solid.

^{1}H NMR (600.1 MHz, CD_2Cl_2, ppm): δ = 12.15 (d, 1H, H1, $J_{P\text{-}H}$ = 20.7 Hz), 8.37 (s, 1H, H5), 7.51 (br, 1H, H3), 7.36 (t, 1H, H8, $J_{P\text{-}H}$ = 4.2 Hz), 6.04 (s, 1H, H10), 4.00 (q, 2H, C*H*$_2$CH$_3$, $J_{H\text{-}H}$ = 7.2 Hz), 3.43 (t, 2H, H12, $J_{P\text{-}H}$ = 4.2 Hz), 1.16 (t, 3H, CH$_2$C*H*$_3$, $J_{H\text{-}H}$ = 7.2 Hz), 0.86 ppm (s, 3H, C*H*$_3$), other peaks at 6.00-8.00 ppm are contributed to protons of phenyl groups. ^{31}P NMR (242.9 MHz, CD_2Cl_2, ppm): δ = 9.25 (s, C2*P*Ph$_3$), -2.26 (s, Os*P*Ph$_3$). ^{13}C NMR (150.9 MHz, CD_2Cl_2, ppm): δ = 231.1 (m, C1), 192.7 (m, C4), 187.7 (s, C9), 176.7 (m, C7), 162.5 (s, *C*=O), 161.1 (s, C5), 159.3 (d, C3, $J_{P\text{-}C}$ = 34.0 Hz), 146.2 (m, C8), 144.2 (s, C11), 139.9 (s, C10), 128.4 (s, C6), 127.5 (m, C2), 59.0 (s, *C*H$_2$CH$_3$), 36.5 (s, C12), 24.6 (s, *C*H$_3$), 13.7 (s, CH$_2$*C*H$_3$), other peaks at 110.0-140.0 ppm are contributed to carbons of phenyl groups. Anal. Calcd (%) for $C_{70}H_{60}BF_4O_2OsP_3$: C, 64.52; H, 4.64; Found: C, 64.46; H, 4.459.

Synthesis of complex **4d**.

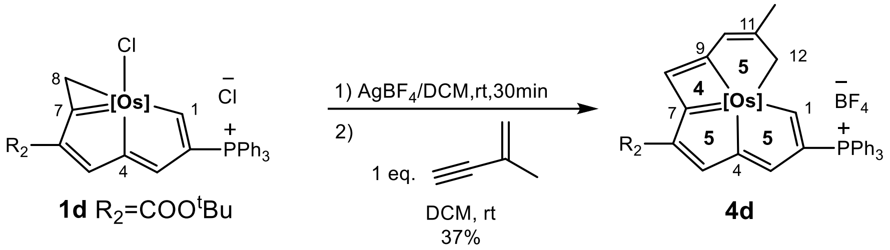

$AgBF_4$ (293 mg, 1.5 mmol) was added into a solution of complex **1d** (625 mg, 0.5 mmol) in CH_2Cl_2 (10 mL) in argon (Ar) atmosphere. The reaction mixture was stirred at room temperature for 30 mins to yield a brown solution mixed with Ag-salt (AgCl) solid. The filtration was then carried out with diatomite to remove AgCl, and a limpid brown solution was obtained, which was added with some additional CH_2Cl_2 (10 mL). Subsequently, about 1.05 eq. of 2-methyl-1-butene-3-yne (about 10 μL) was added dropwise into the solution in argon (Ar) atmosphere. After stirring at room temperature for 1 hour, a brown solution was generated. This solution was reduced to about 3 mL under vacuum, in which ethyl ether (20 mL) was next added to give some precipitate. The precipitate was collected by filtration and washed with ethyl ether (2 × 20 mL), affording **4d** (248 mg, 37% yield) as a brown solid.

^1H NMR (600.1 MHz, CD_2Cl_2, ppm): δ = 12.12 (d, 1H, H1, J_{P-H} = 19.8 Hz), 8.34 (s, 1H, H5), 7.52 (s, 1H, H3), 7.28 (t, 1H, H8, J_{P-H} = 3.6 Hz), 6.03 (s, 1H, H10), 3.42 (t, 2H, H12, J_{P-H} = 6.0 Hz), 1.3 (s, 9H, C(C*H*$_3$)$_3$), 0.85 (s, 3H, C11C*H*$_3$), other peaks at 6.00-8.00 ppm are contributed to protons of phenyl groups. ^{31}P NMR (242.9 MHz, CD_2Cl_2, ppm): δ = 9.22 (s, C2*P*Ph$_3$), -2.31 (s, Os*P*Ph$_3$). ^{13}C NMR (150.9 MHz, CD_2Cl_2, ppm): δ = 230.5 (m, C1), 192.0 (m, C4), 187.4 (s, C9), 178.3 (m, C7), 161.6 (s, *C*=O), 161.1 (s, C5), 158.7 (d, C3, J_{P-C} = 24.0 Hz), 146.1 (m, C8), 146.0 (s, C11), 139.7 (s, C10), 127.8 (s, C6), 127.7 (m, C2), 79.0 (s, *C*(CH$_3$)$_3$), 36.4 (s, C12), 27.4 (s, C(*C*H$_3$)$_3$), 24.4 (s, C11*C*H$_3$), other peaks at 110.0-140.0 ppm are contributed to carbons of phenyl groups. Anal. Calcd (%) for $C_{72}H_{64}BF_4O_2OsP_3$: C, 64.96; H, 4.85; Found: C, 65.05; H, 4.495.

4. Other Supplementary Figures and Tables

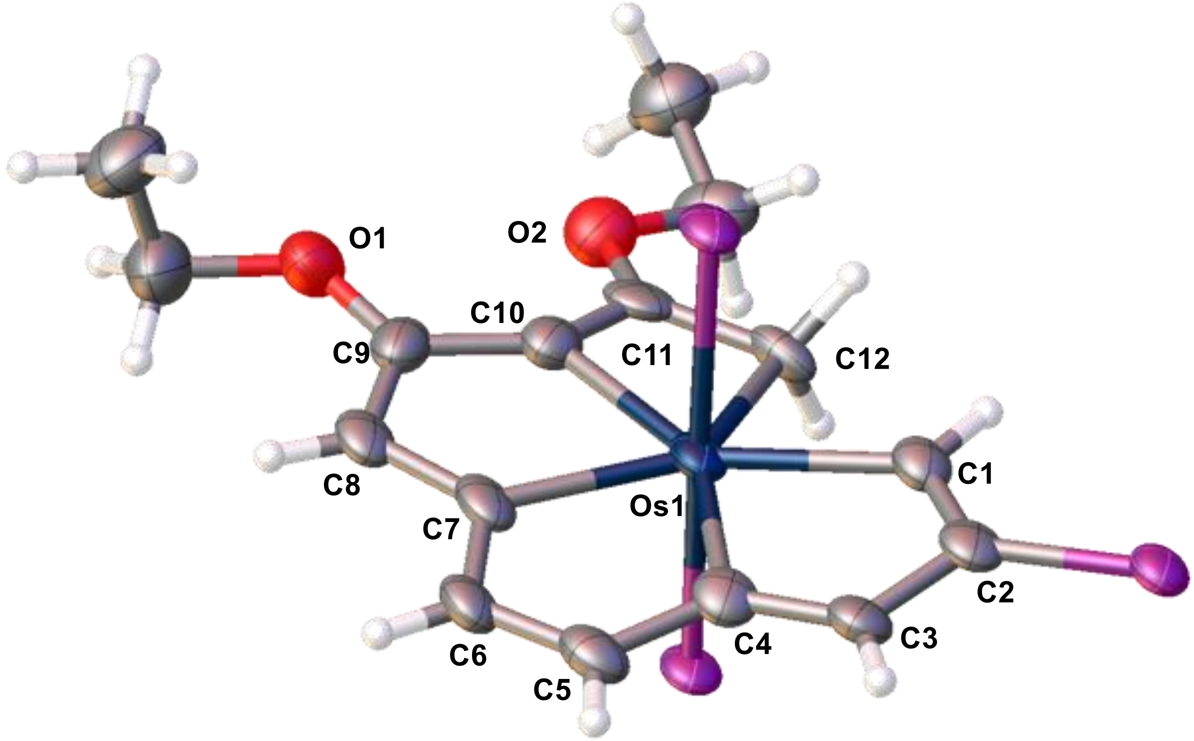

Figure S9. X-ray molecular structure for the cation of complex **3a** drawn with 50% probability level. The phenyl groups in PPh_3 are omitted for clarity. Selected bond lengths [Å] and angles [°]: Os1–C1 2.096(5), Os1–C4 2.173(6), Os1–C7 2.112(5), Os1–C10 2.119(6), Os1–C12 2.278(5), C1–C2 1.367(8), C2–C3 1.435(8), C3–C4 1.332(9), C4–C5 1.434(9), C5–C6 1.361(9), C6–C7 1.434(9) , C7–C8 1.398(9), C8–C9 1.390(9), C9–C10 1.413(8), C10–C11 1.377(9), C11–C12 1.436(9); Os1–C1–C2 119.3(4), C1–C2–C3 114.3(5), C2–C3–C4 115.1(5), C3–C4–Os1 117.5(5), C1–Os1–C4 73.8(2), Os1–C4–C5 116.0(4), C4–C5–C6 115.3(5), C5–C6–C7 116.0(5), C6–C7–Os1 117.7(4), C7–Os1–C4 74.7(2), Os1–C7–C8 121.1(4), C7–C8–C9 113.5(5), C8–C9–C10 112.4(5), C9–C10–Os1 120.8(4), C7–Os1–C10 72.0(2), Os1–C10–C11 101.0(4), C10–C11–C12 107.2(5), C11–C12–Os1 90.9(3), C10–Os1–C12 60.9(2).

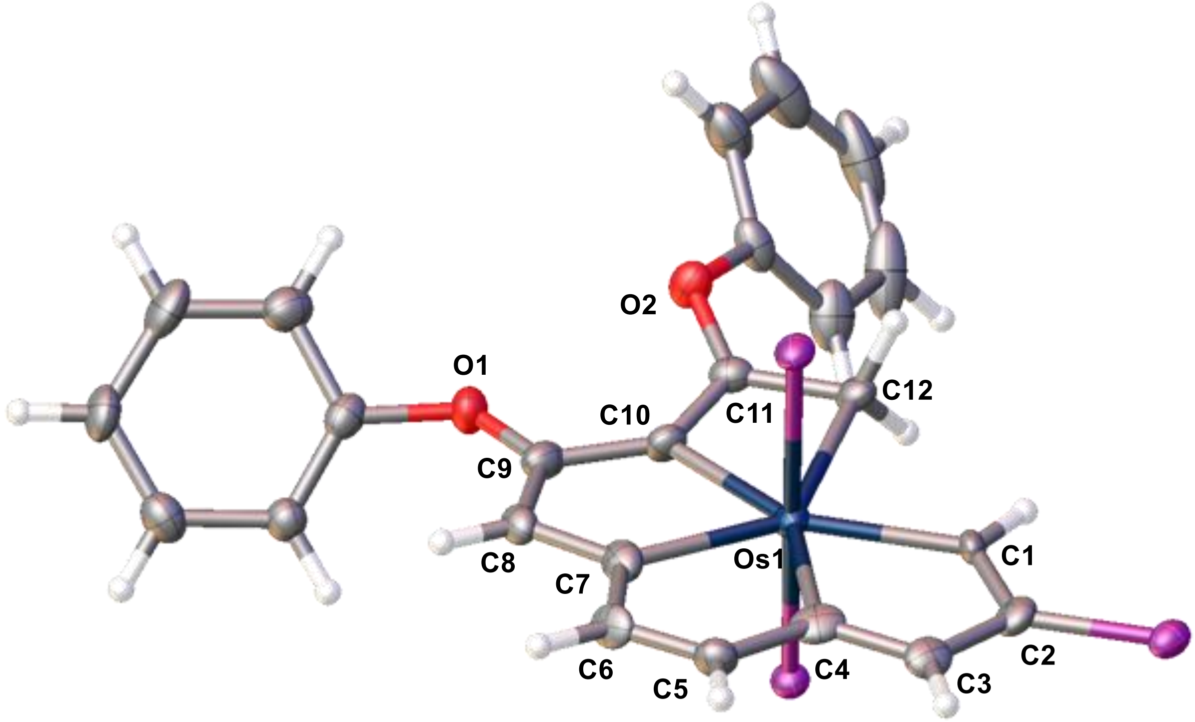

Figure S10. X-ray molecular structure for the cation of complex **3b** drawn with 50% probability level. The phenyl groups in PPh_3 are omitted for clarity. Selected bond lengths [Å] and angles [°]: Os1–C1 2.080(8), Os1–C4 2.133(10), Os1–C7 2.099(10), Os1–C10 2.114(9), Os1–C12 2.269(9), C1–C2 1.358(14), C2–C3 1.433(15), C3–C4 1.352(15), C4–C5 1.421(15), C5–C6 1.346(15), C6–C7 1.436(15) , C7–C8 1.407(14), C8–C9 1.345(14), C9–C10 1.419(14), C10–C11 1.313(14), C11–C12 1.496(14); Os1–C1–C2 120.1(7), C1–C2–C3 114.1(9), C2–C3–C4 113.2(9), C3–C4–Os1 118.9(8), C1–Os1–C4 73.5(4), Os1–C4–C5 117.5(7), C4–C5–C6 115.1(9), C5–C6–C7 114.7(9), C6–C7–Os1 118.4(7), C7–Os1–C4 74.1(4), Os1–C7–C8 122.2(7), C7–C8–C9 112.5(9), C8–C9–C10 112.7(8), C9–C10–Os1 121.0(7), C7–Os1–C10 71.5(4), Os1–C10–C11 102.3(7), C10–C11–C12 106.1(8), C11–C12–Os1 89.9(6), C10–Os1–C12 61.6(4).

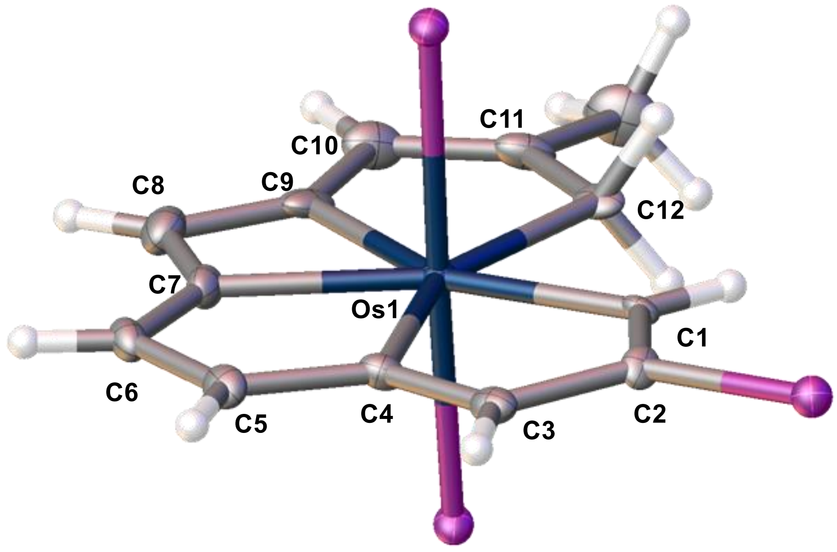

Figure S11. X-ray molecular structure for the cation of complex **4a** drawn with 50% probability level. The phenyl groups in PPh_3 are omitted for clarity. Selected bond lengths [Å] and angles [°]: Os–C1 2.070(8), Os–C4 2.120(8), Os–C7 2.074(8), Os–C9 2.129(8), Os–C12 2.274(8), C1–C2 1.398(11), C2–C3 1.436(12), C3–C4 1.361(12), C4–C5 1.440(12), C5–C6 1.361(12), C6–C7 1.425(12) , C7–C8 1.396(14), C8–C9 1.382(14), C9–C10 1.429(15), C10–C11 1.349(14), C11–C12 1.503(14), C11–C13 1.508(14); Os–C1–C2 119.9(5), C1–C2–C3 112.9(7), C2–C3–C4 113.7(8), C3–C4–Os 119.2(6), C1–Os–C4 74.2(3), Os–C4–C5 117.9(6), C4–C5–C6 114.9(8), C5–C6–C7 112.0(8), C6–C7–Os 121.8(6), C7–Os–C4 73.3(3), Os–C7–C8 102.3(6), C7–C8–C9 97.8(8), C8–C9–Os 100.2(7), C7–Os–C9 59.7(4), C9–C10–C11 112.5(8), C10–C11–C12 117.5(8), C11–C12–Os 113.8(6), C9–Os–C12 73.3(4).

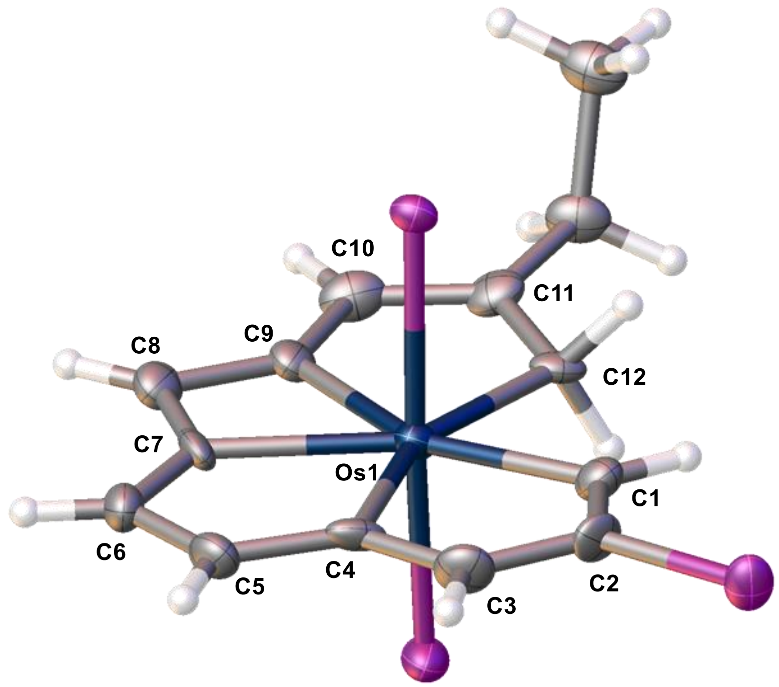

Figure S12. X-ray molecular structure for the cation of complex **4b** drawn with 50% probability level. The phenyl groups in PPh_3 are omitted for clarity. Selected bond lengths [Å] and angles [°]: Os–C1 2.067(18), Os–C4 2.135(14), Os–C7 2.096(14), Os–C9 2.133(14), Os–C12 2.203(12), C1–C2 1.376 (19), C2–C3 1.38(2), C3–C4 1.39(3), C4–C5 1.40(2), C5–C6 1.37(2), C6–C7 1.449(19), C7–C8 1.34(2), C8–C9 1.41(2), C9–C10 1.415(17), C10–C11 1.367(16), C11–C12 1.477(15), C11–C13 1.498(12); Os–C1–C2 120.1(11), C1–C2–C3 113.7(14), C2–C3–C4 116.0(13), C3–C4–Os 115.8(12), C1–Os–C4 74.4(6), Os–C4–C5 119.8(12), C4–C5–C6 115.0(12), C5–C6–C7 111.6(13), C6–C7–Os 121.1(11), C7–Os–C4 72.4(6), Os–C7–C8 104.0(10), C7–C8–C9 97.4(15), C8–C9–Os 100.0(10), C7–Os–C9 58.5(5), C9–C10–C11 114.0(10), C10–C11–C12 114.1(9), C11–C12–Os 117.0(9), C9–Os–C12 72.0(5).

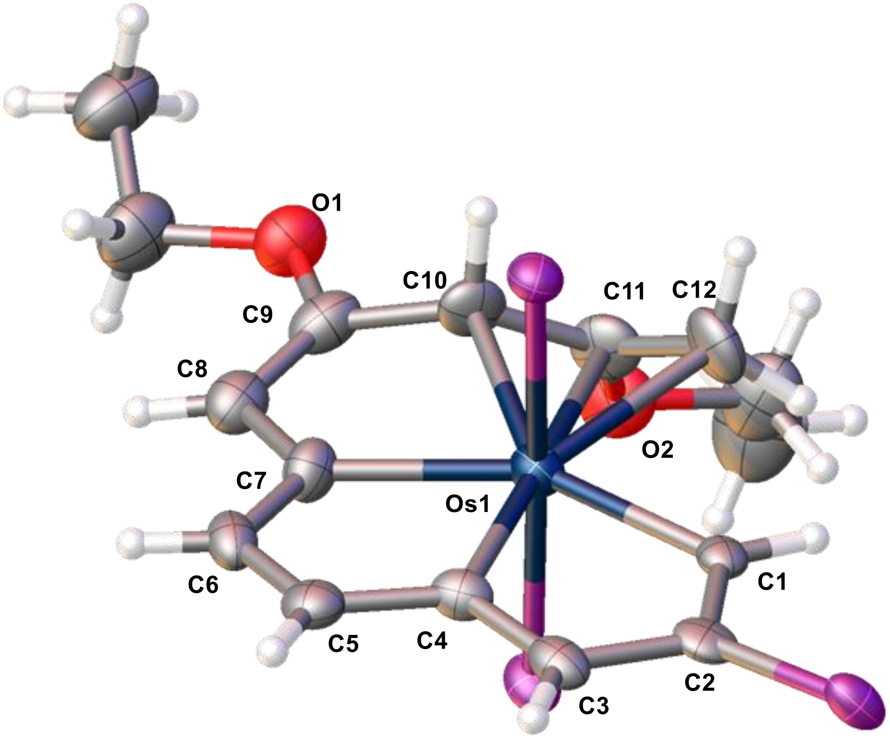

Figure S13. X-ray molecular structure for the cation of complex **5a** drawn with 50% probability level. The phenyl groups in PPh_3 are omitted for clarity. Selected bond lengths [Å] and angles [°]: Os–C1 2.125(5), Os–C4 2.128(5), Os–C7 2.113(6), Os–C10 2.249(6), Os–C11 2.450(6), Os–C12 2.320(6), C1–C2 1.345(8), C2–C3 1.433(8), C3–C4 1.361(9), C4–C5 1.397(9), C5–C6 1.351(9), C6–C7 1.412(9) , C7–C8 1.426(9), C8–C9 1.361(10), C9–C10 1.460(10), C10–C11 1.400(10), C11–C12 1.392(10); Os–C1–C2 118.6(4), C1–C2–C3 115.3(5), C2–C3–C4 113.6(5), C3–C4–Os 118.7(4), C1–Os–C4 73.8(2), Os–C4–C5 116.8(4), C4–C5–C6 116.2(6), C5–C6–C7 114.9(5), C6–C7–Os 117.5(4), C7–Os–C4 74.3(2), Os–C7–C8 122.4(5), C7–C8–C9 113.2(6), C8–C9–C10 115.0(6), C9–C10–Os 114.4(4), C7–Os–C10 71.4(2), Os–C10–C11 80.7(4), C10–C11–C12 113.5(6), C11–C12–Os 78.1(3), C10–Os–C12 61.3(2).

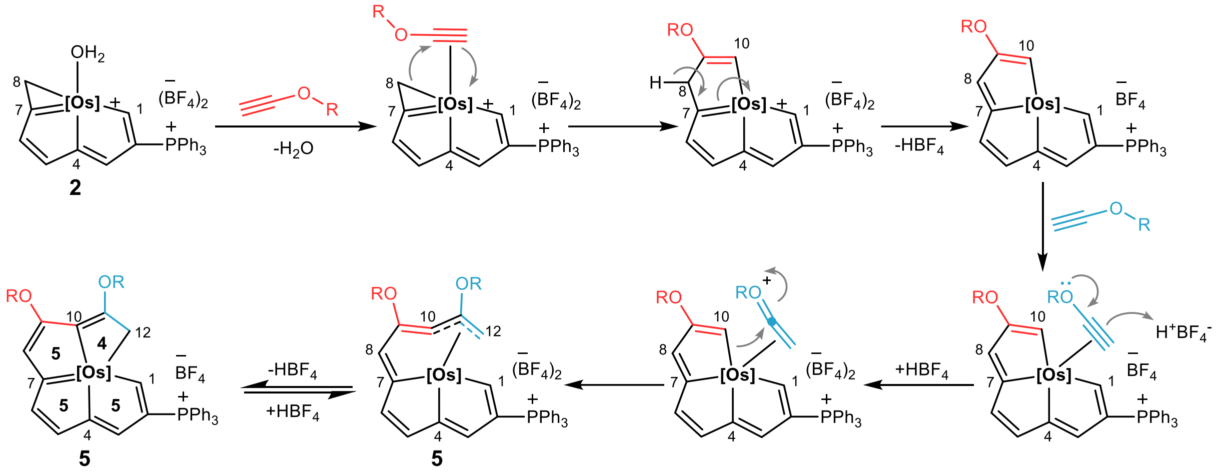

Figure S14. Plausible mechanisms for the formation of complexes **3**.

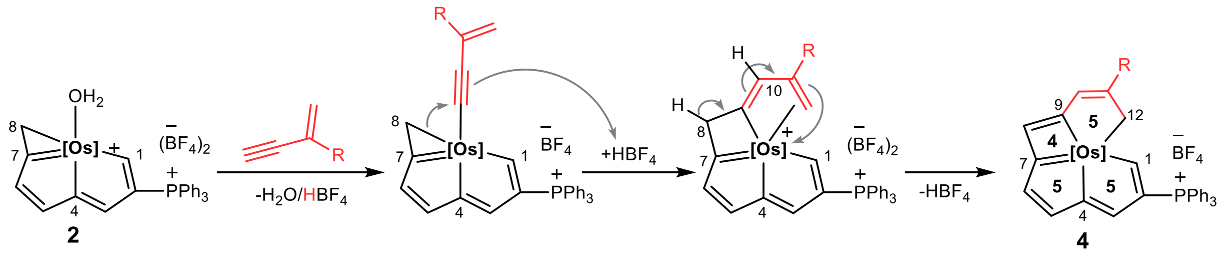

Figure S15. Plausible mechanisms for the formation of complexes **4**.

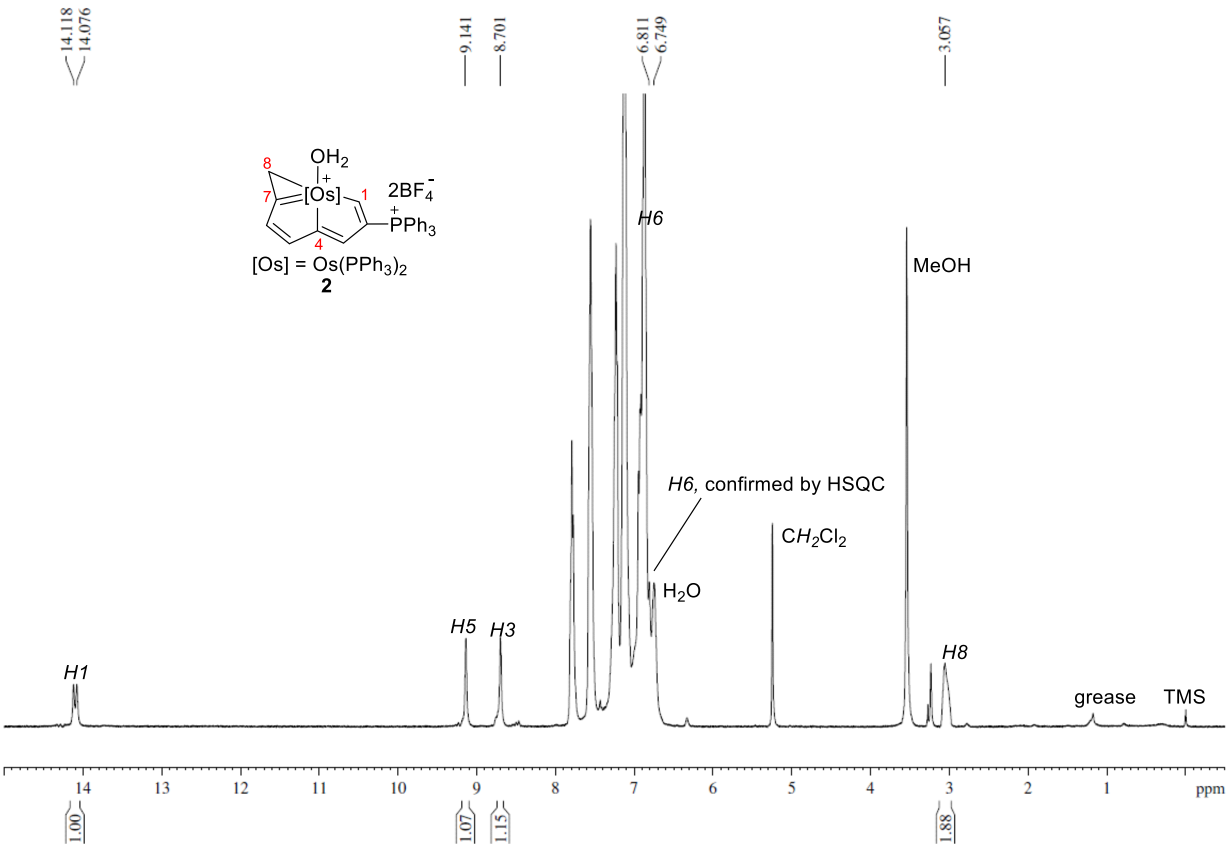

Figure S16. ^{1}H-NMR spectrum (400.0 MHz) of **2** in CD_2Cl_2 with 10% CD_3OD.

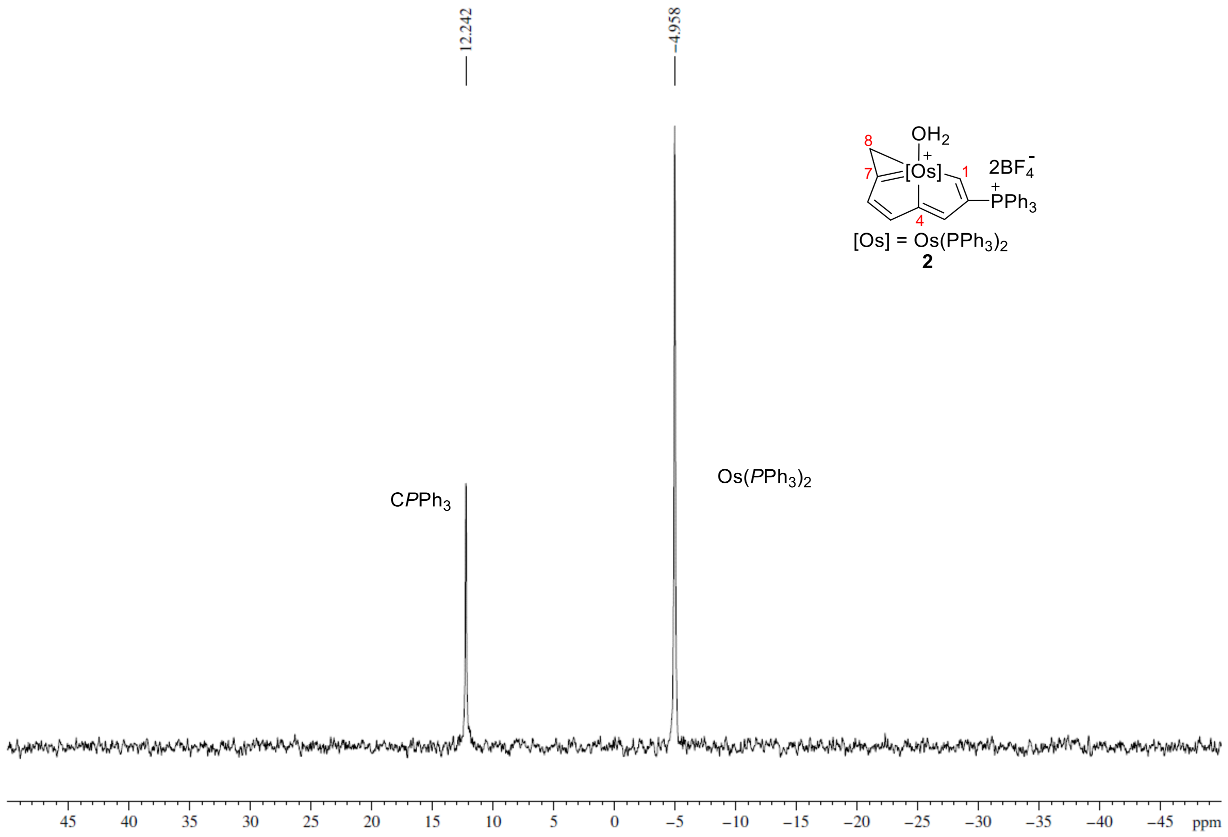

Figure S17. ^{31}P-NMR spectrum (161.9 MHz) of **2** in CD_2Cl_2 with 10% CD_3OD.

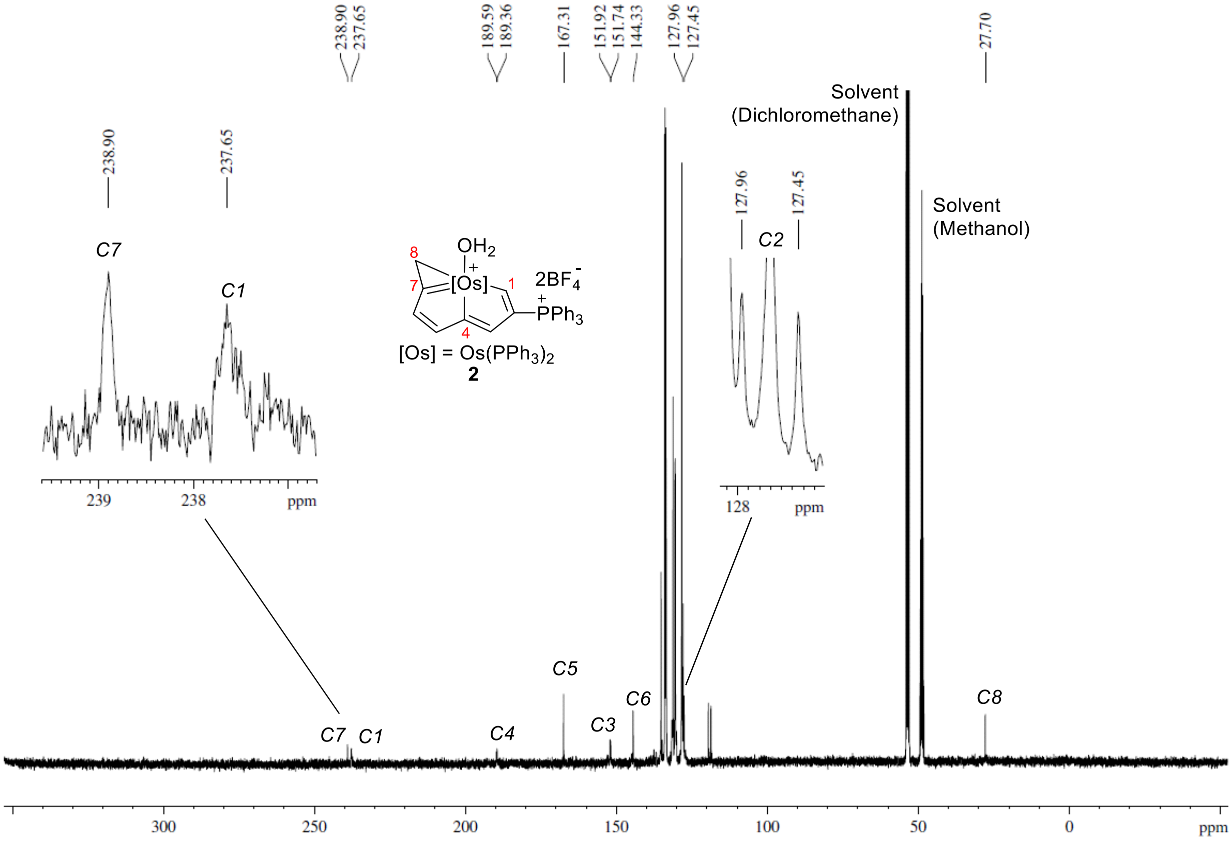

Figure S18. ^{13}C-NMR spectrum (100.6 MHz) of **2** in CD_2Cl_2 with 10% CD_3OD.

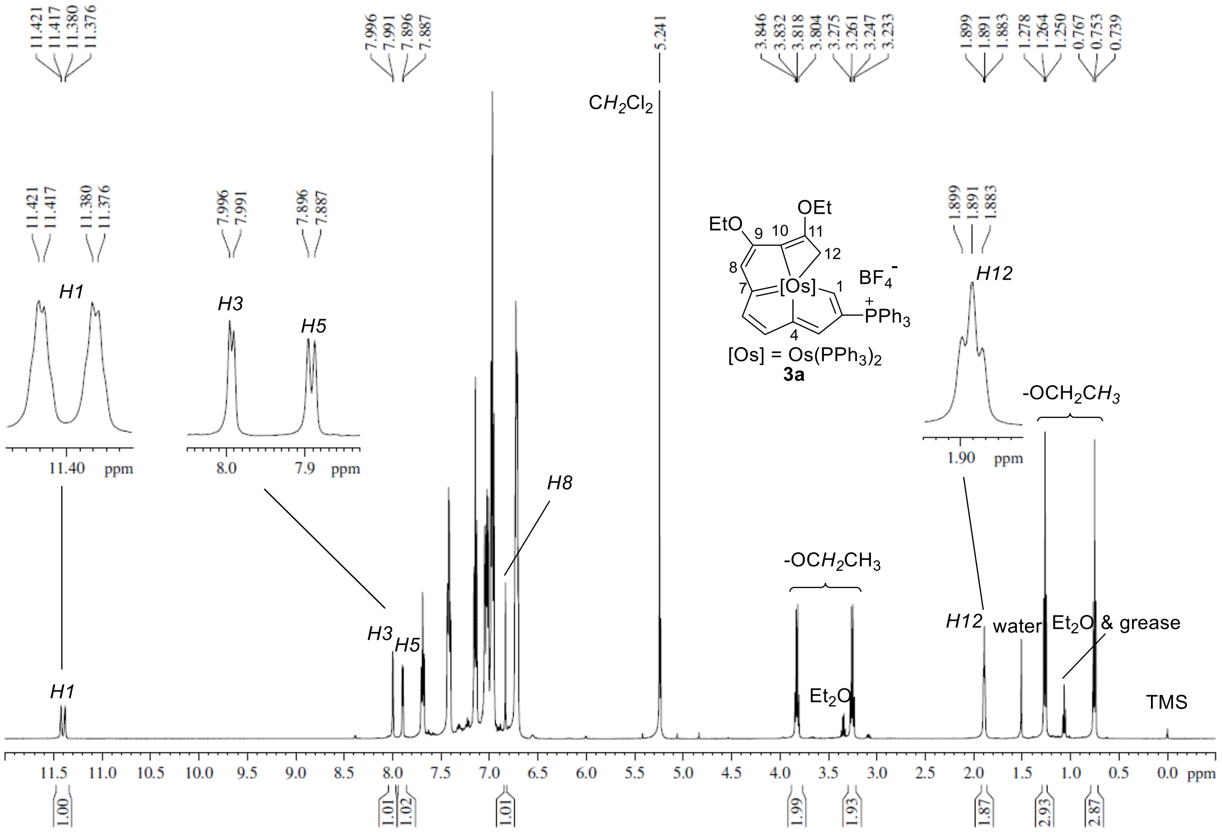

Figure S19. ^{1}H-NMR spectrum (400.0 MHz) of **3a** in CD_2Cl_2.

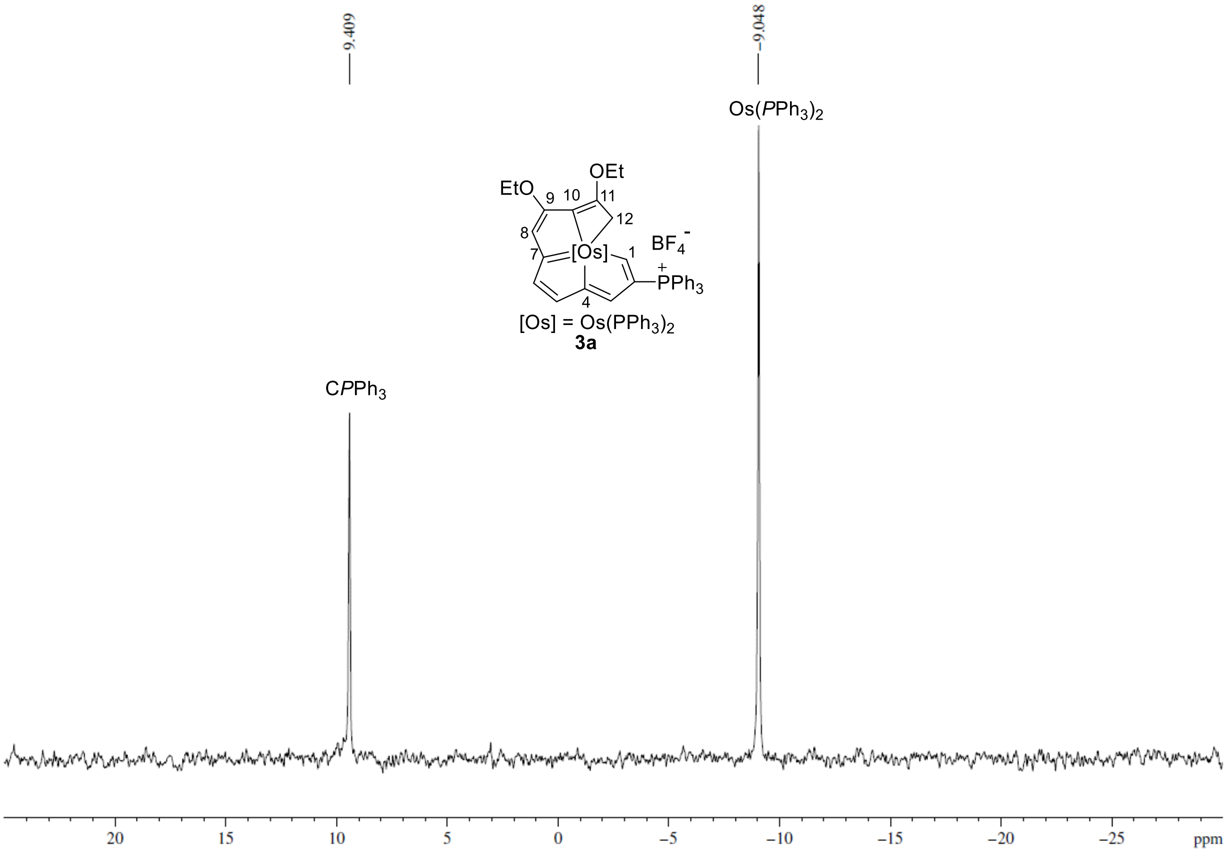

Figure S20. ^{31}P-NMR spectrum (161.9 MHz) of **3a** in CD_2Cl_2.

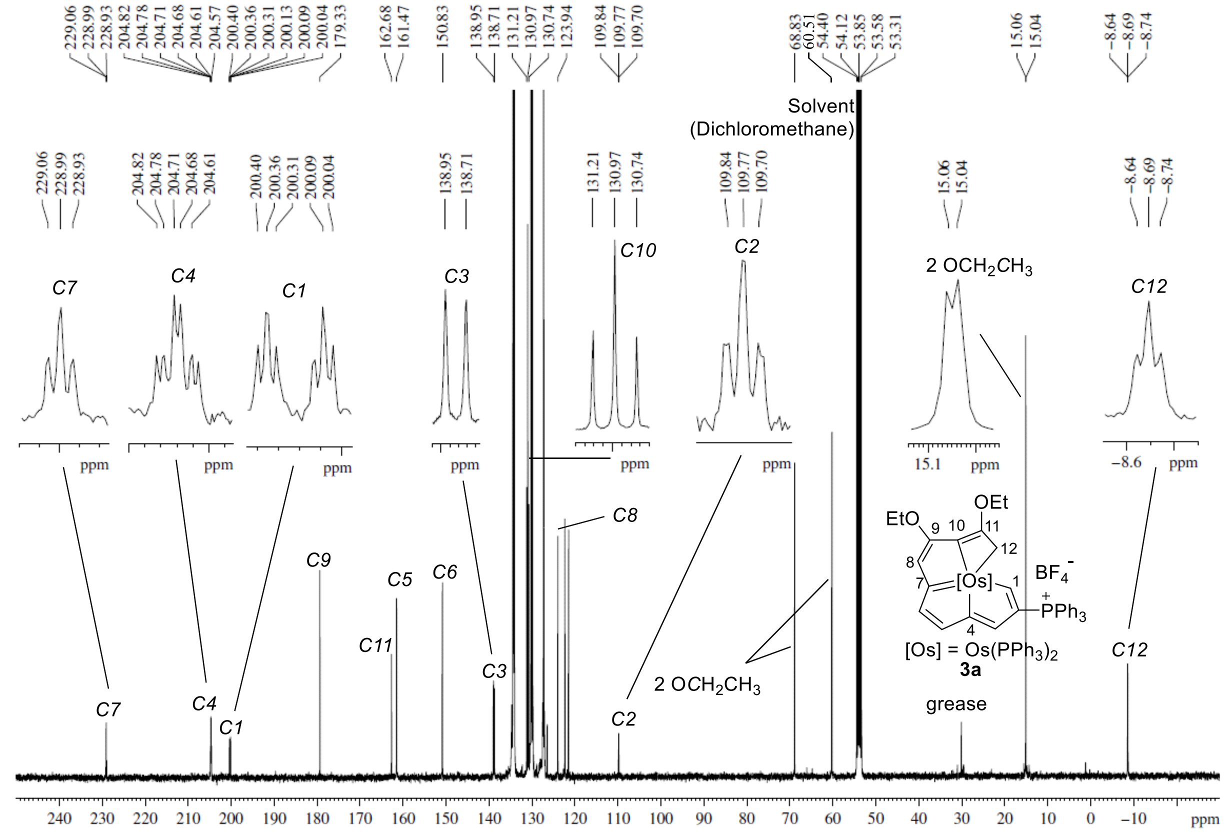

Figure S21. ^{13}C-NMR spectrum (100.6 MHz) of **3a** in CD_2Cl_2.

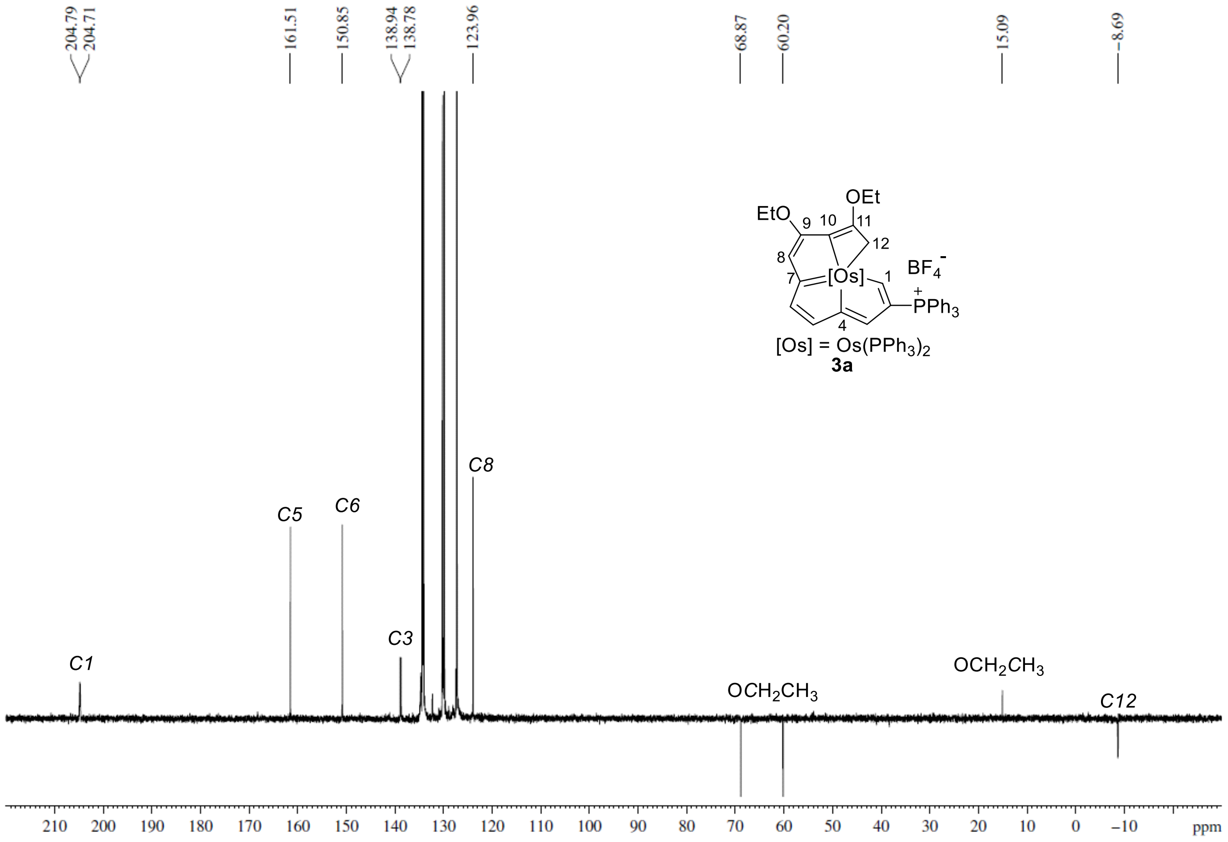

Figure S22. ^{13}C-DEPT-135 spectrum (100.6 MHz) of **3a** in CD_2Cl_2.

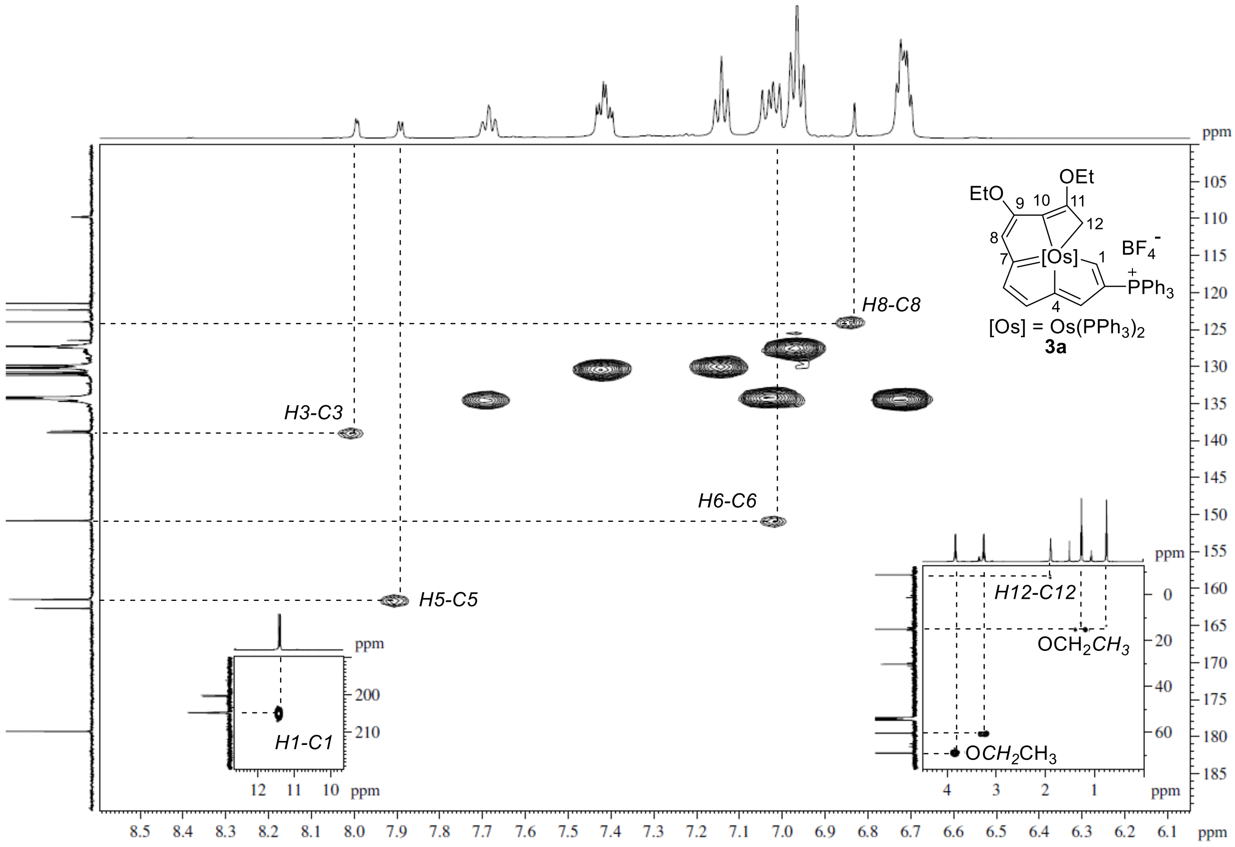

Figure S23. Two-dimensional ^{1}H-^{13}C-HSQC spectrum of **3a** in CD_2Cl_2.

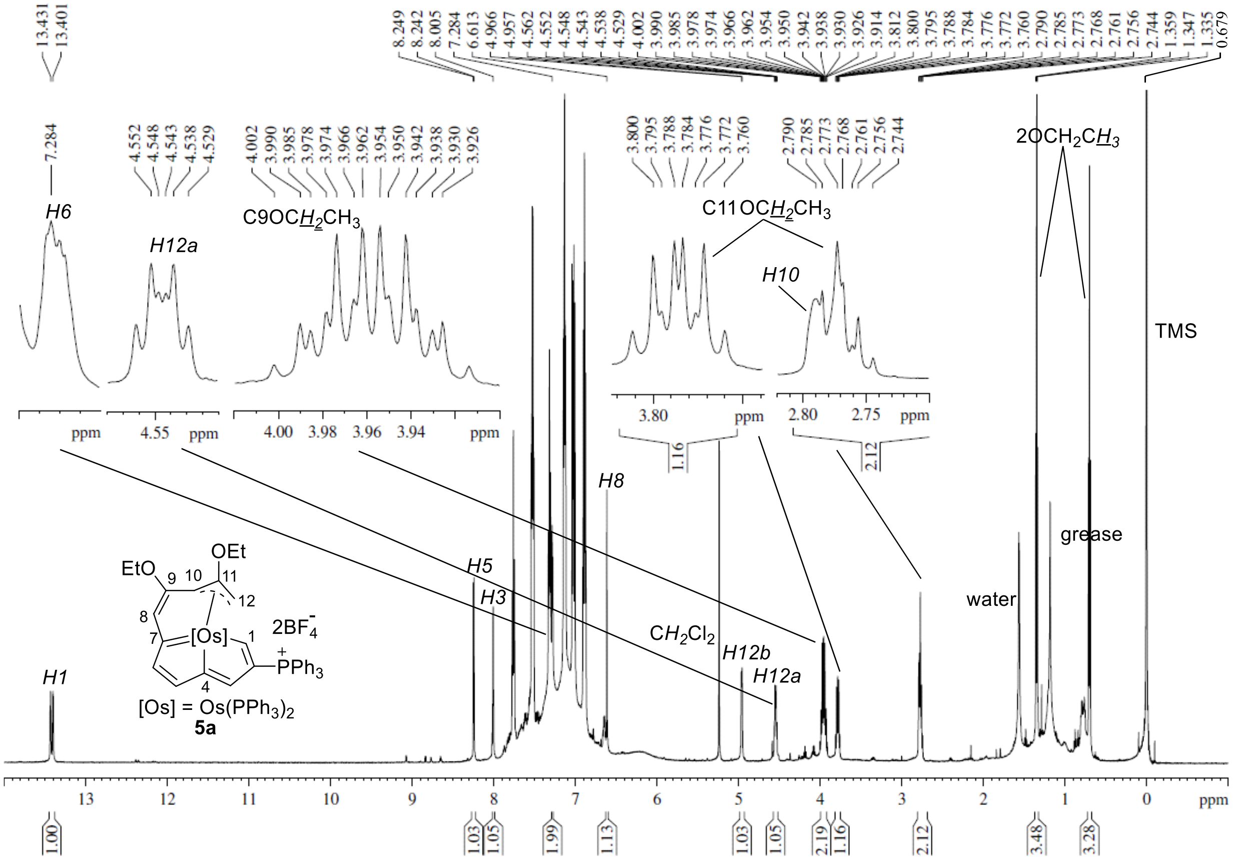

Figure S24. ^{1}H-NMR spectrum (600.1 MHz) of **5a** in CD_2Cl_2.

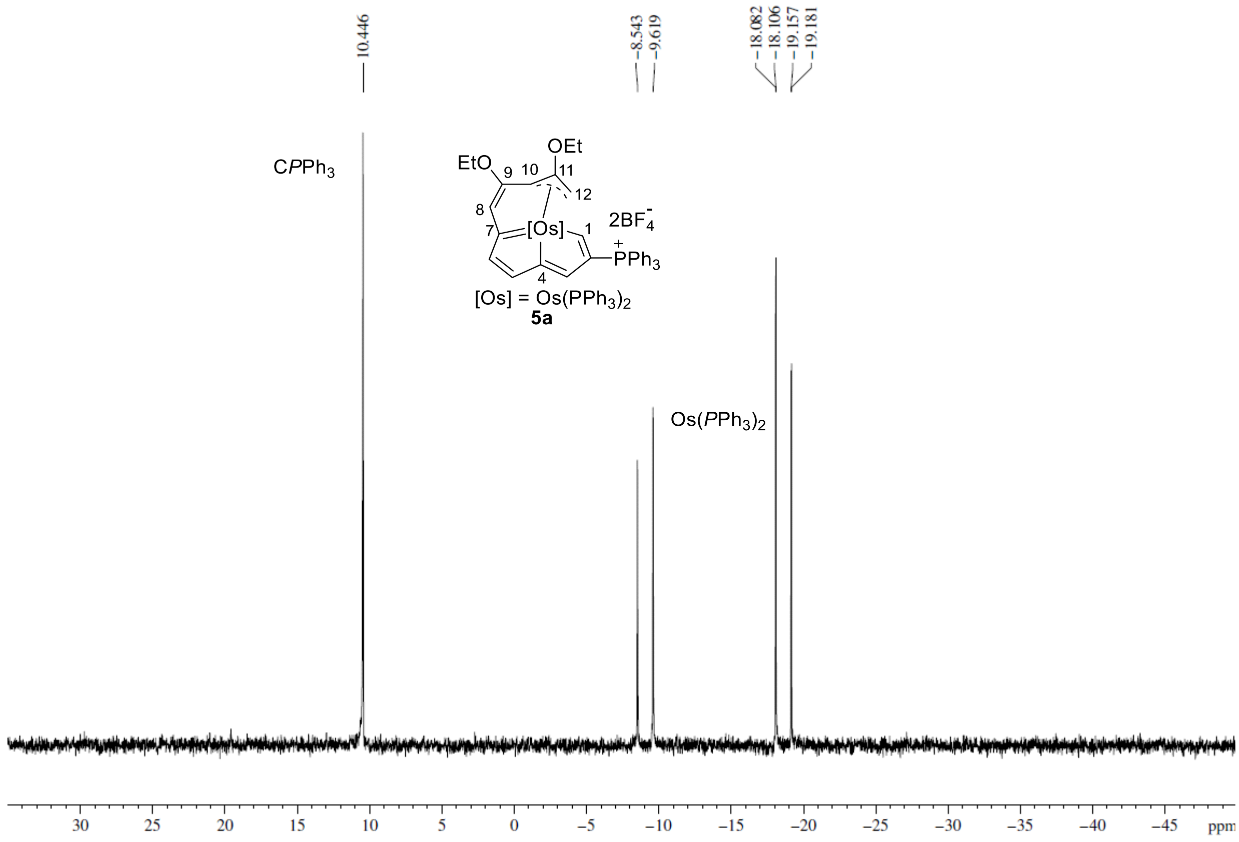

Figure S25. ^{31}P-NMR spectrum (242.9 MHz) of **5a** in CD_2Cl_2.

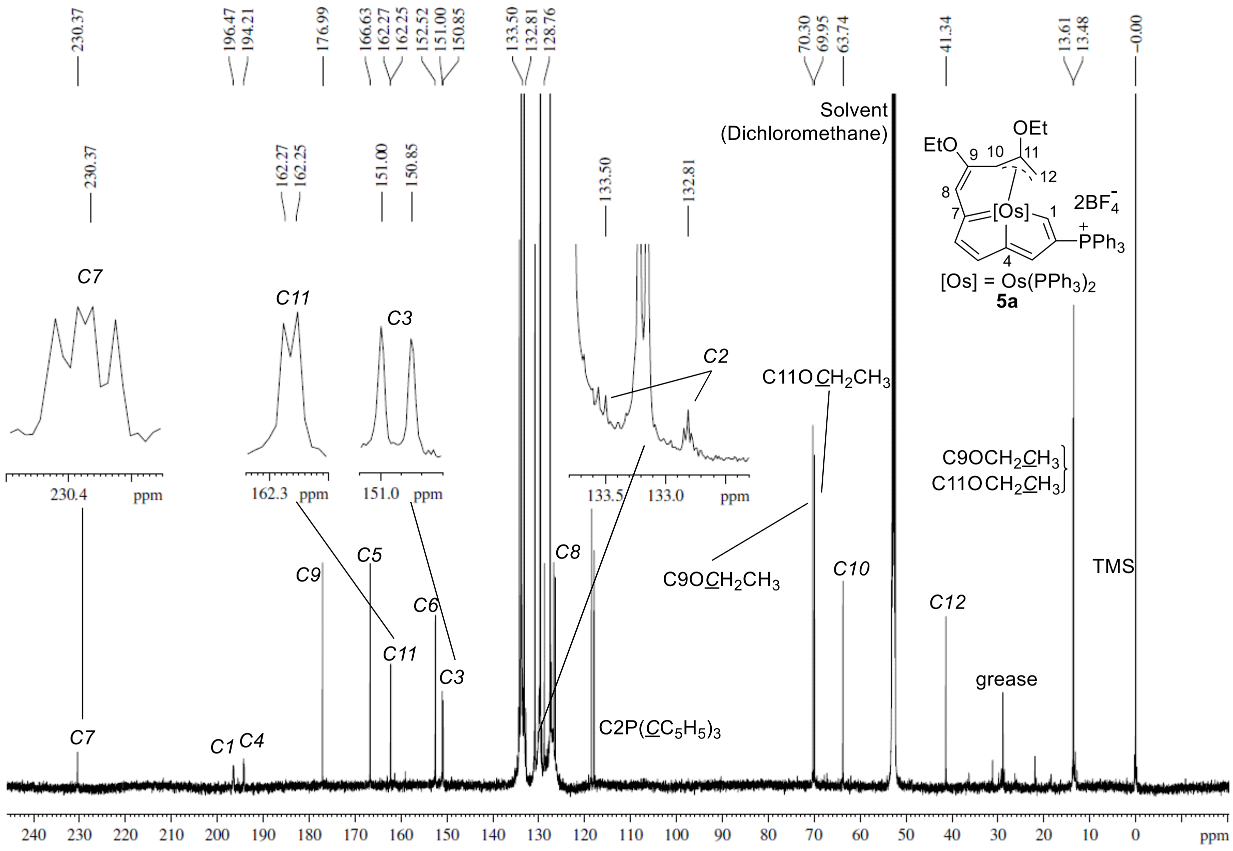

Figure S26. ^{13}C-NMR spectrum (150.9 MHz) of **5a** in CD_2Cl_2.

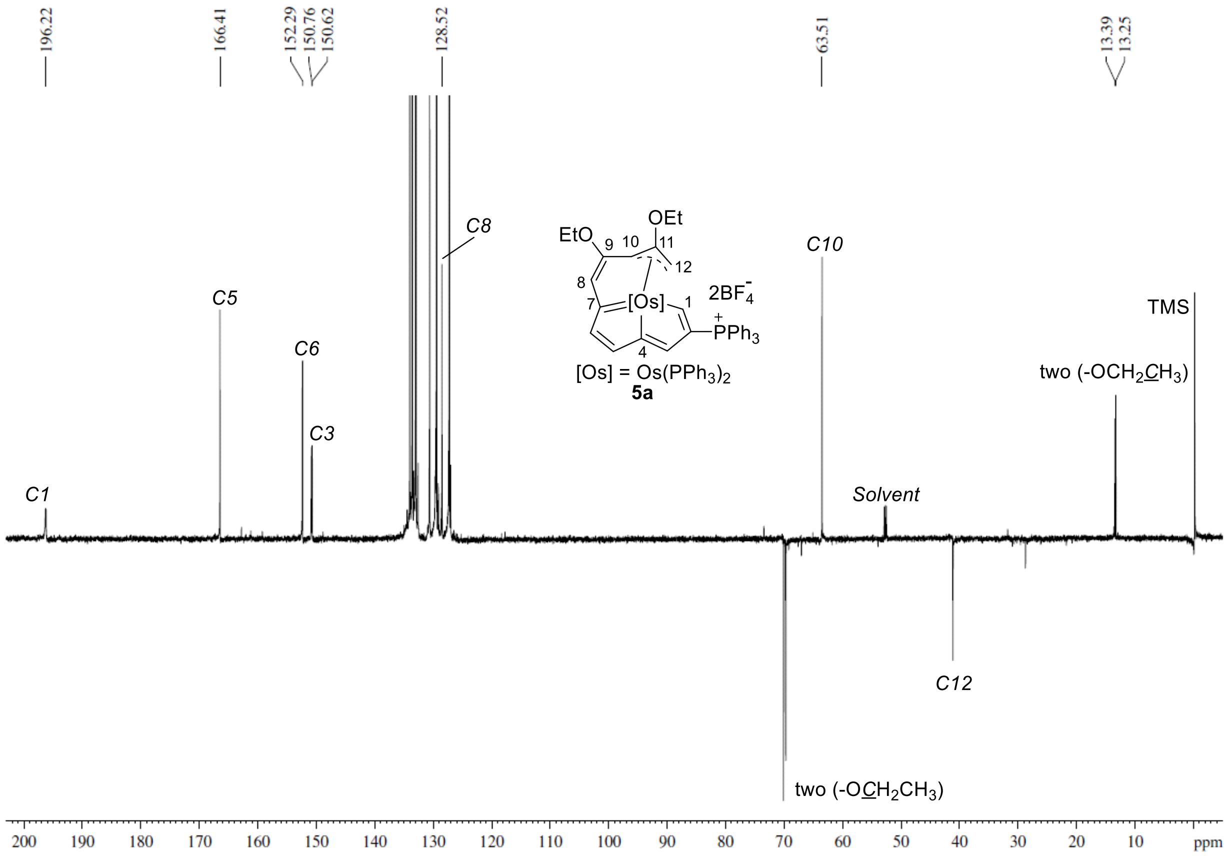

Figure S27. ^{13}C-DEPT-135 spectrum (150.9 MHz) of **5a** in CD_2Cl_2.

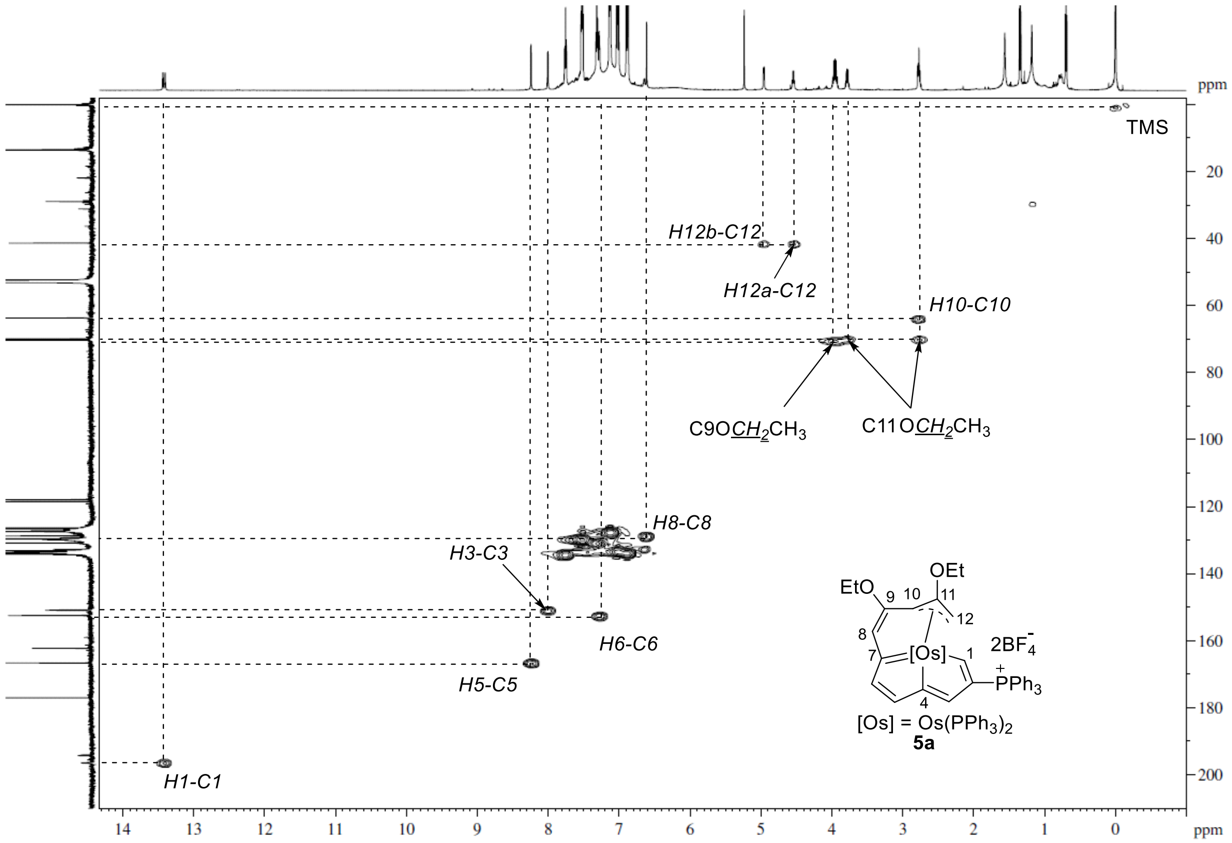

Figure S28. Two-dimensional ^{1}H-^{13}C-HSQC spectrum of **5a** in CD_2Cl_2.

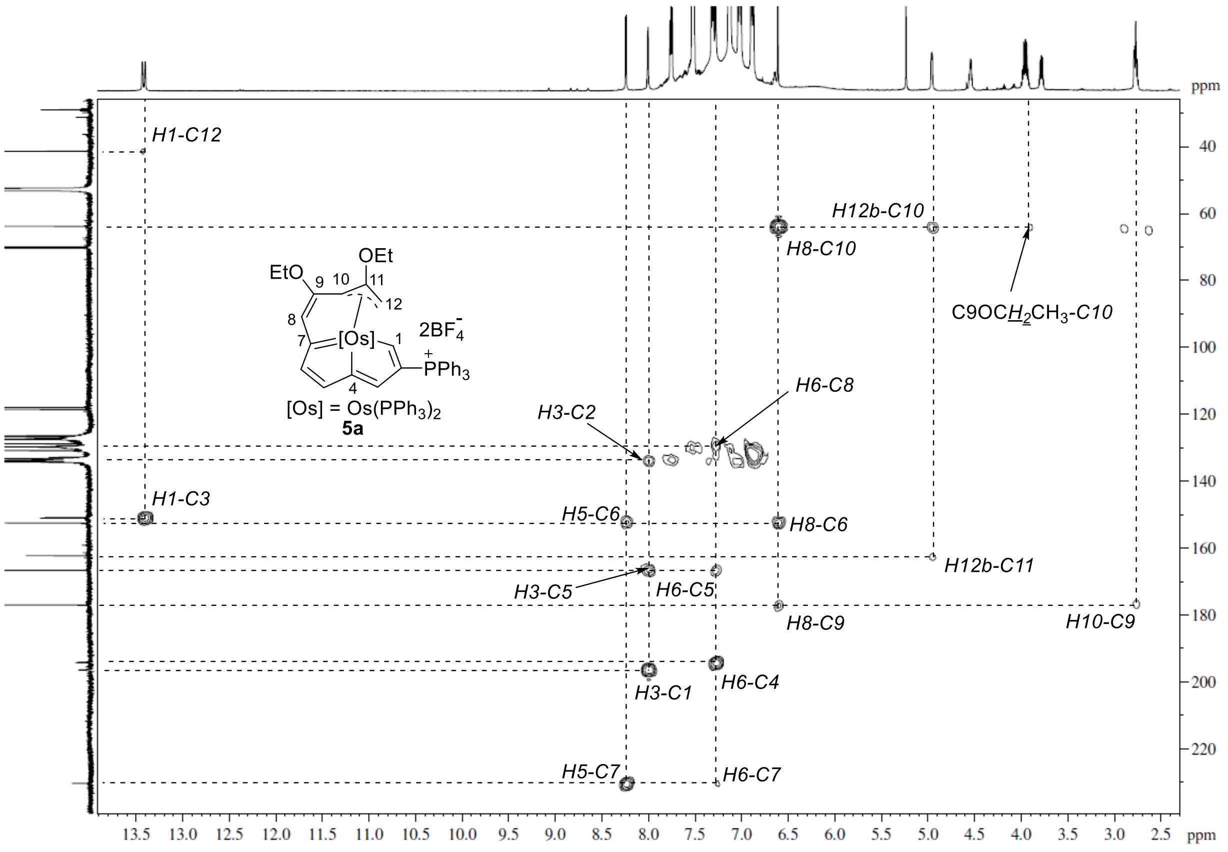

Figure S29. Two-dimensional ^1H-^{13}C-HMBC spectrum of **5a** in CD_2Cl_2.

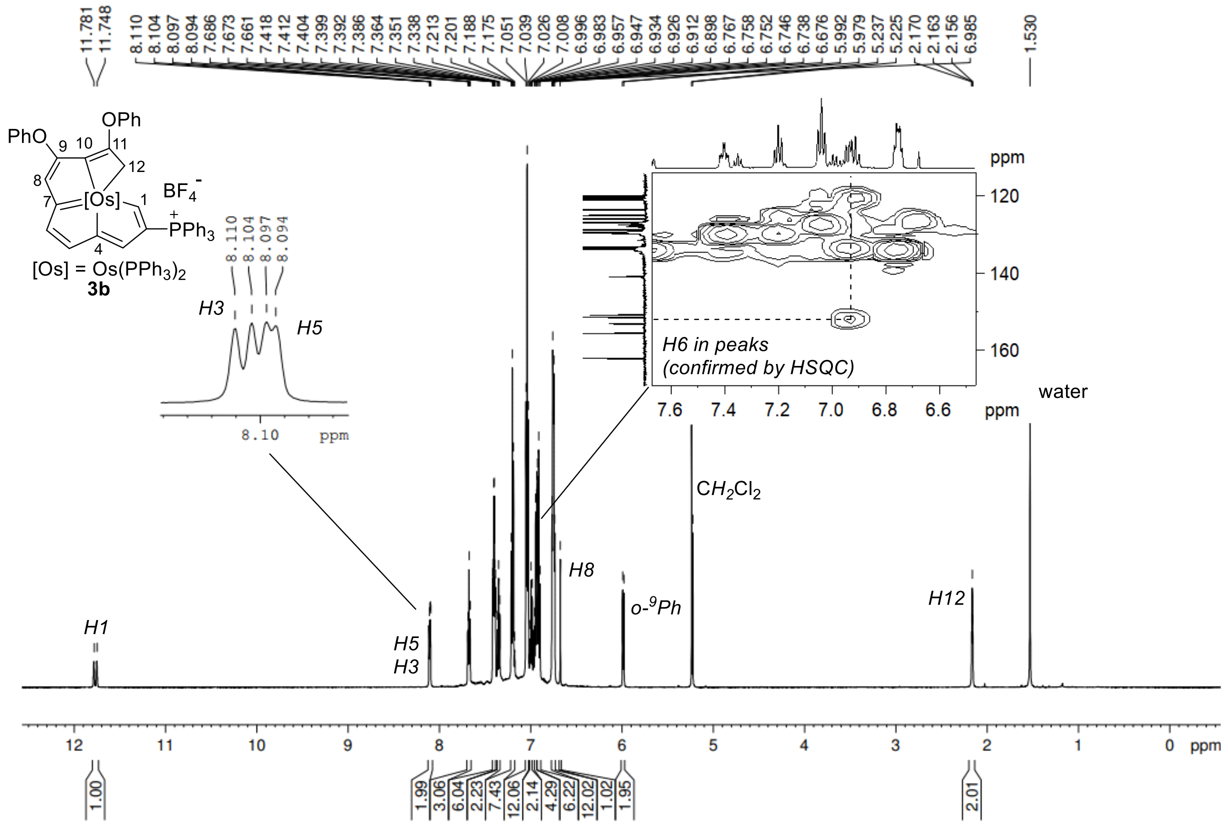

Figure S30. ^{1}H-NMR spectrum (600.1 MHz) of **3b** in CD_2Cl_2.

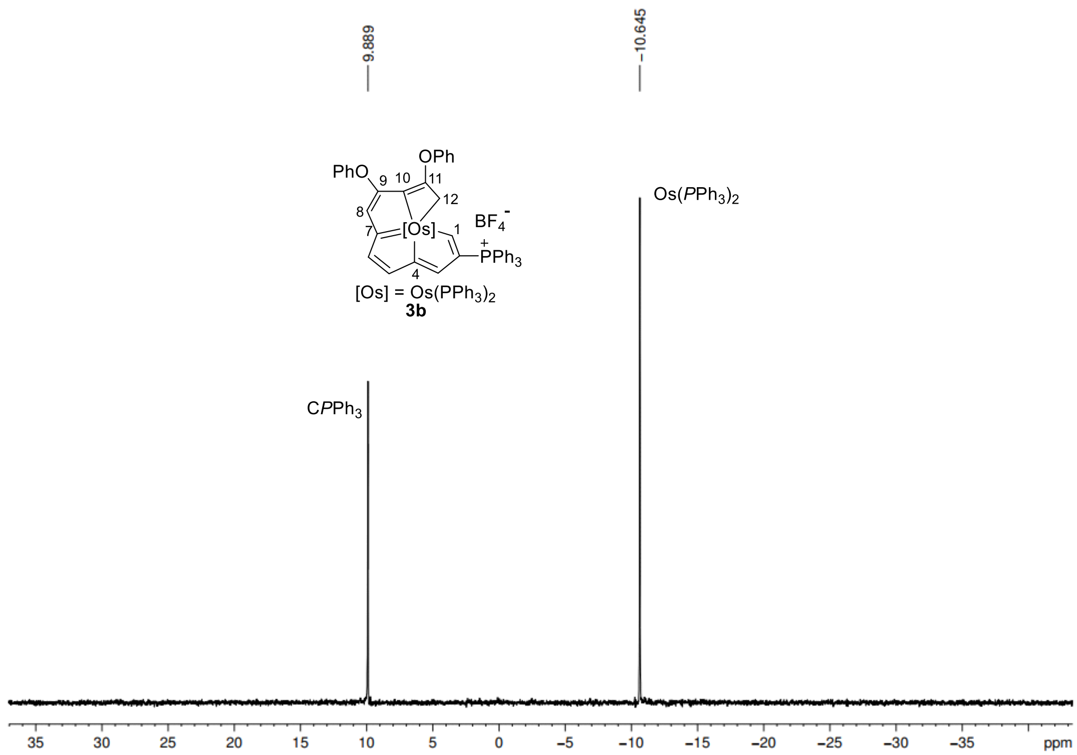

Figure S31. ^{31}P-NMR spectrum (161.9 MHz) of **3b** in CD_2Cl_2.

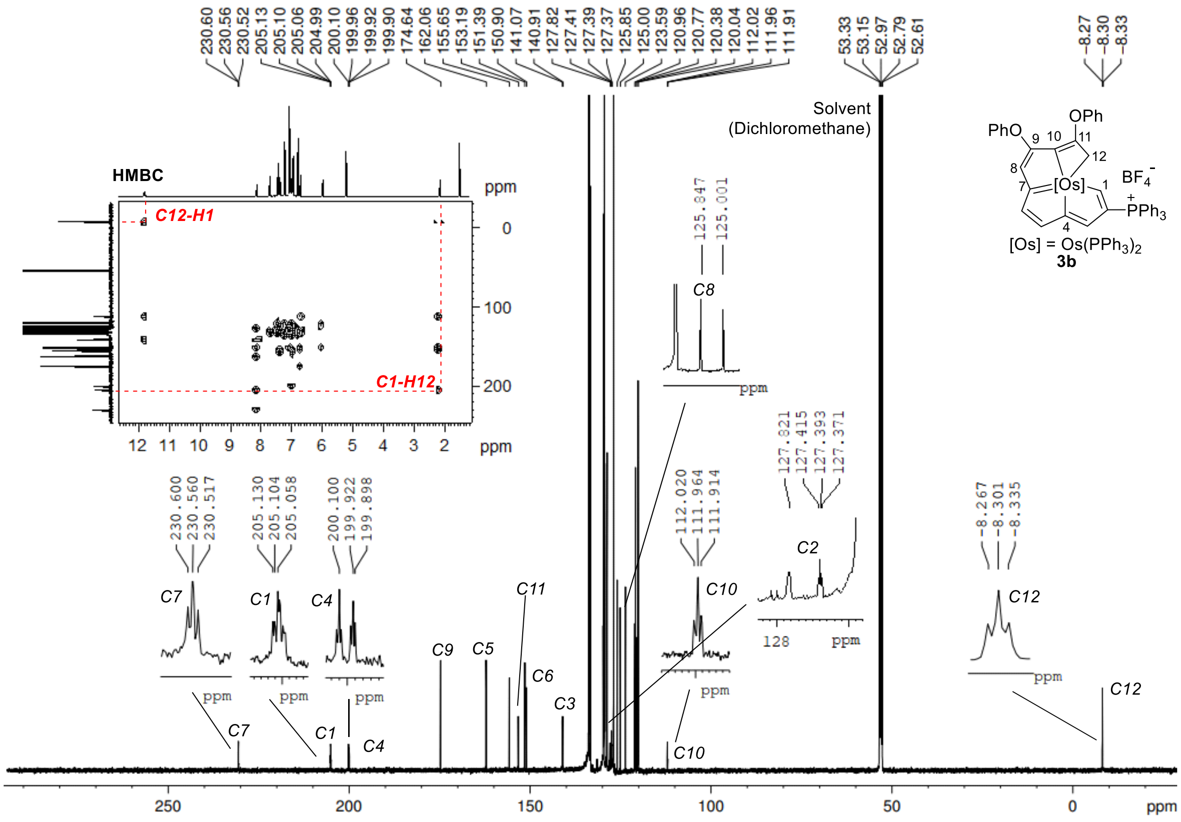

Figure S32. ^{13}C-NMR spectrum (100.6 MHz) of **3b** in CD_2Cl_2 (with HMBC).

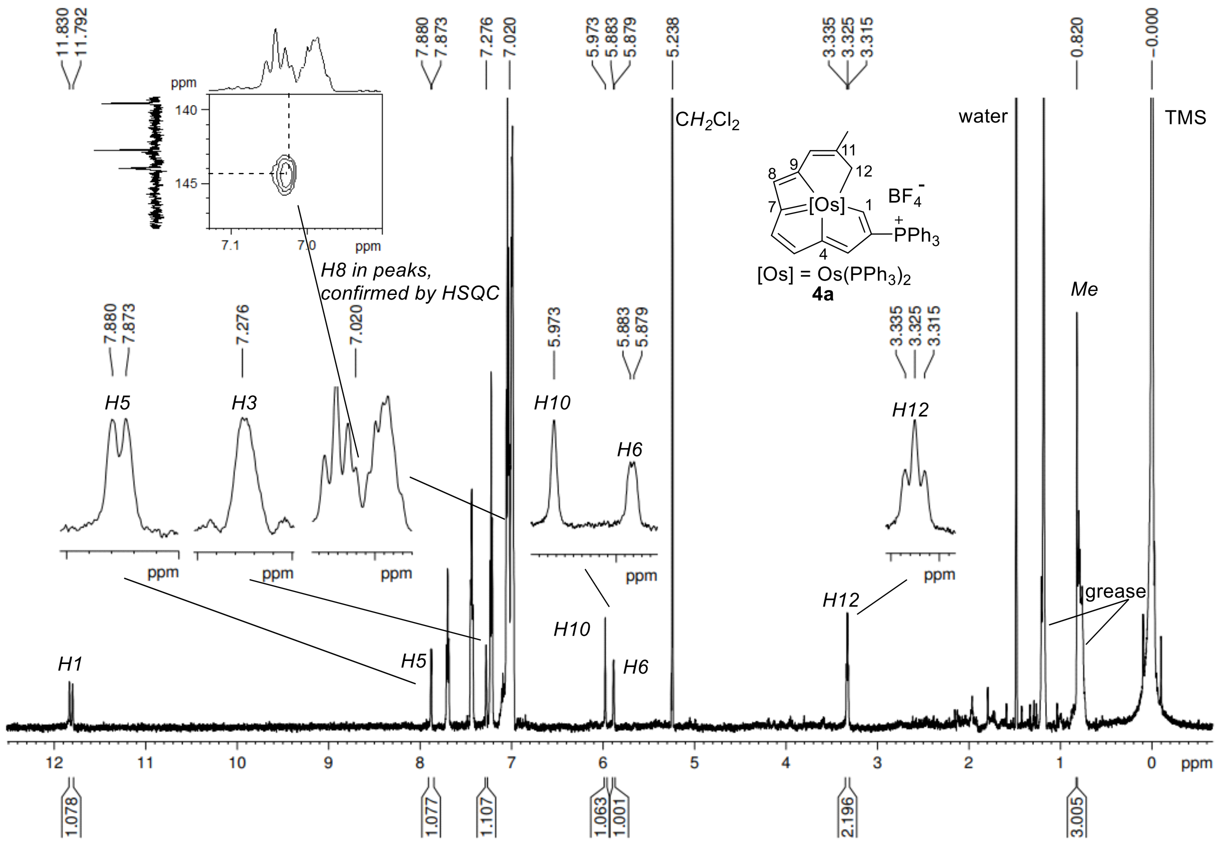

Figure S33. ^{1}H-NMR spectrum (600.1 MHz) of **4a** in CD_2Cl_2.

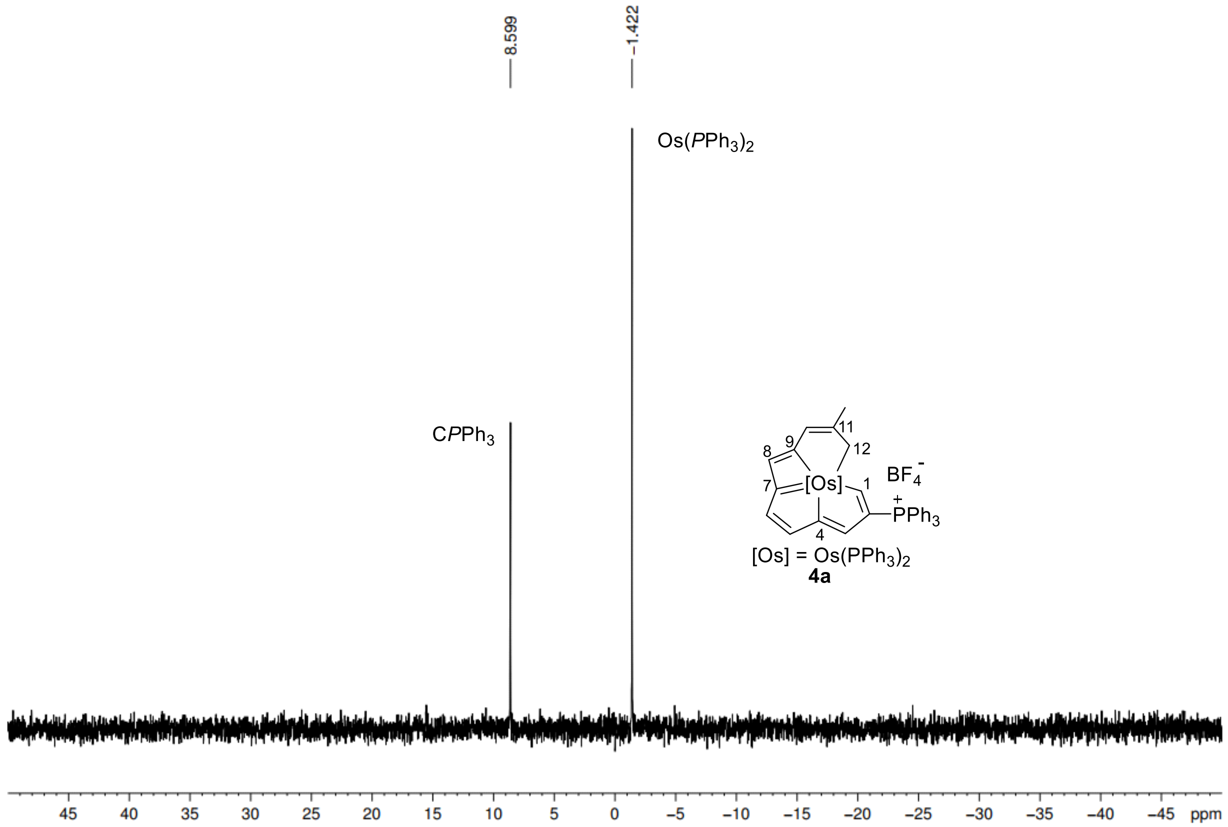

Figure S34. ^{31}P-NMR spectrum (242.9 MHz) of **4a** in CD_2Cl_2.

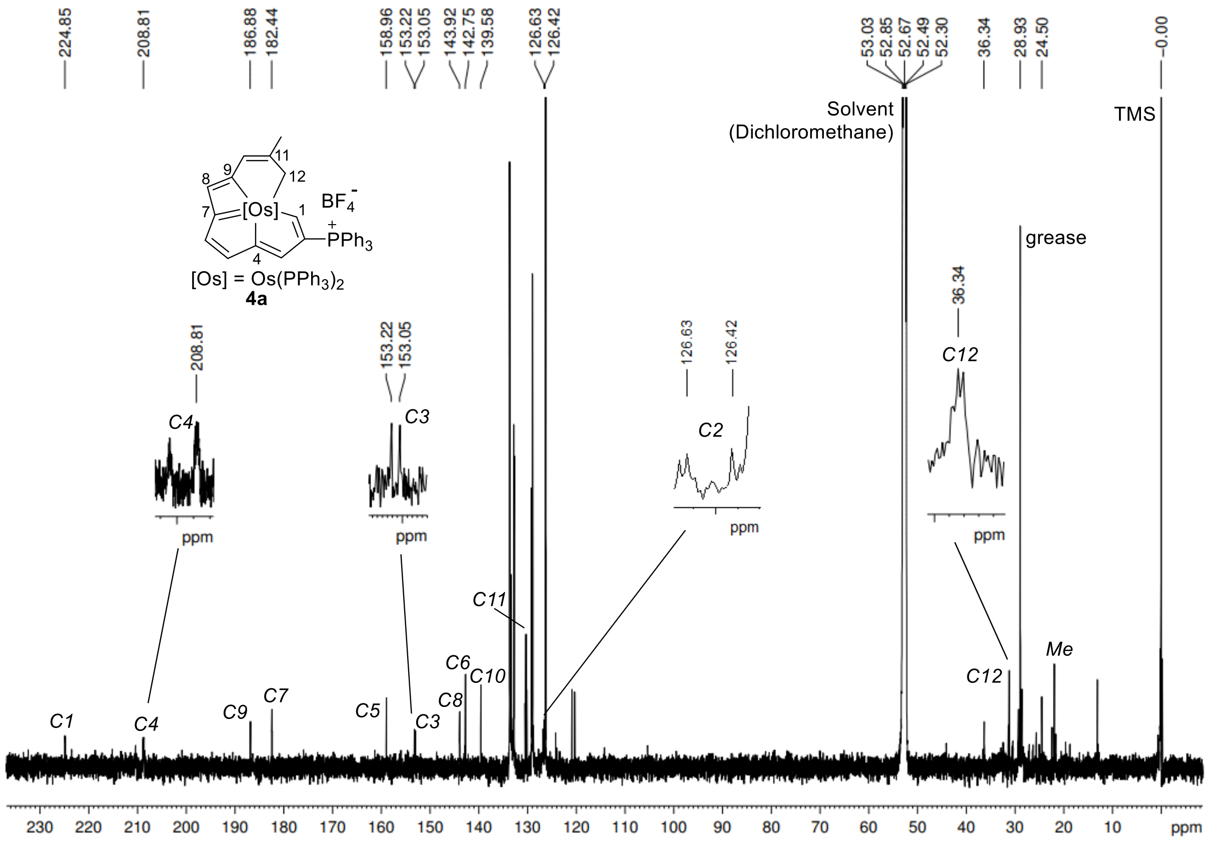

Figure S35. ^{13}C-NMR spectrum (150.9 MHz) of **4a** in CD_2Cl_2.

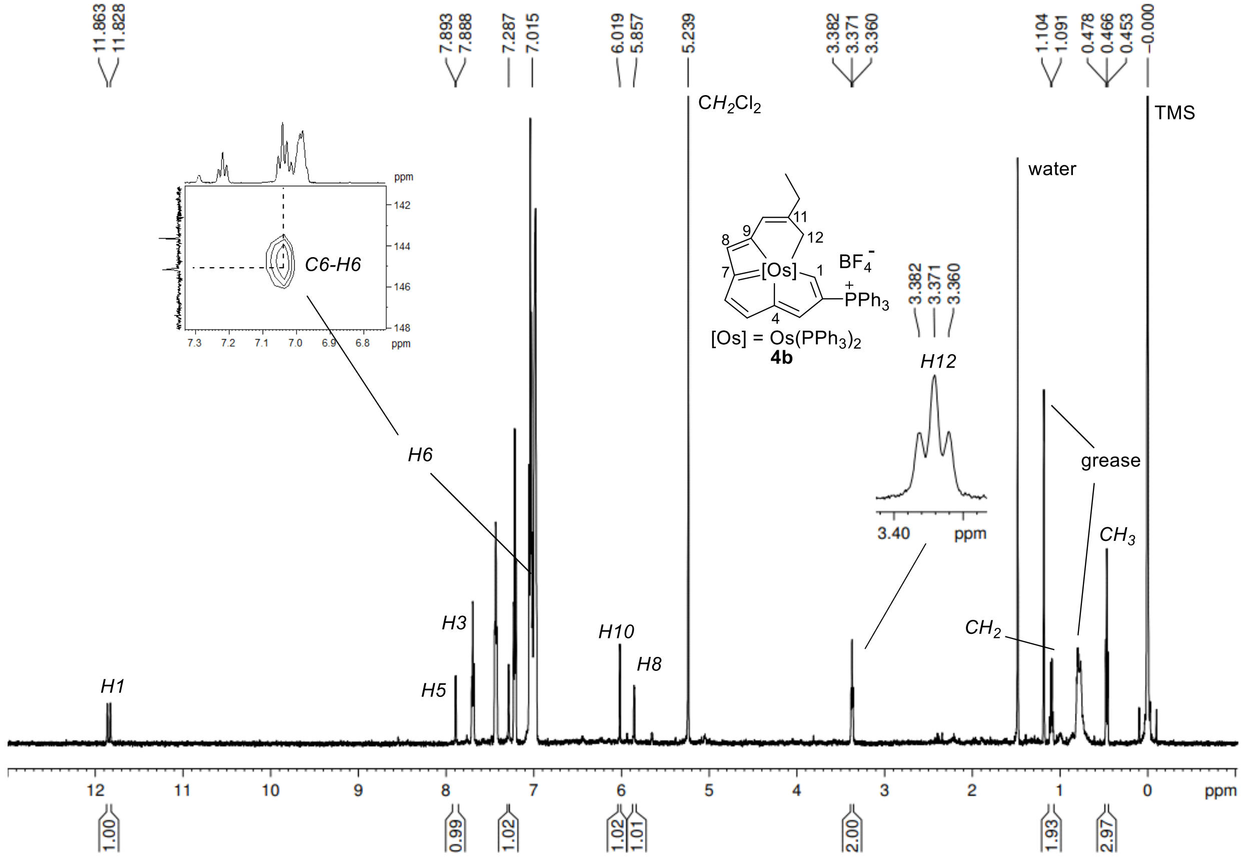

Figure S36. ^{1}H-NMR spectrum (600.1 MHz) of **4b** in CD_2Cl_2.

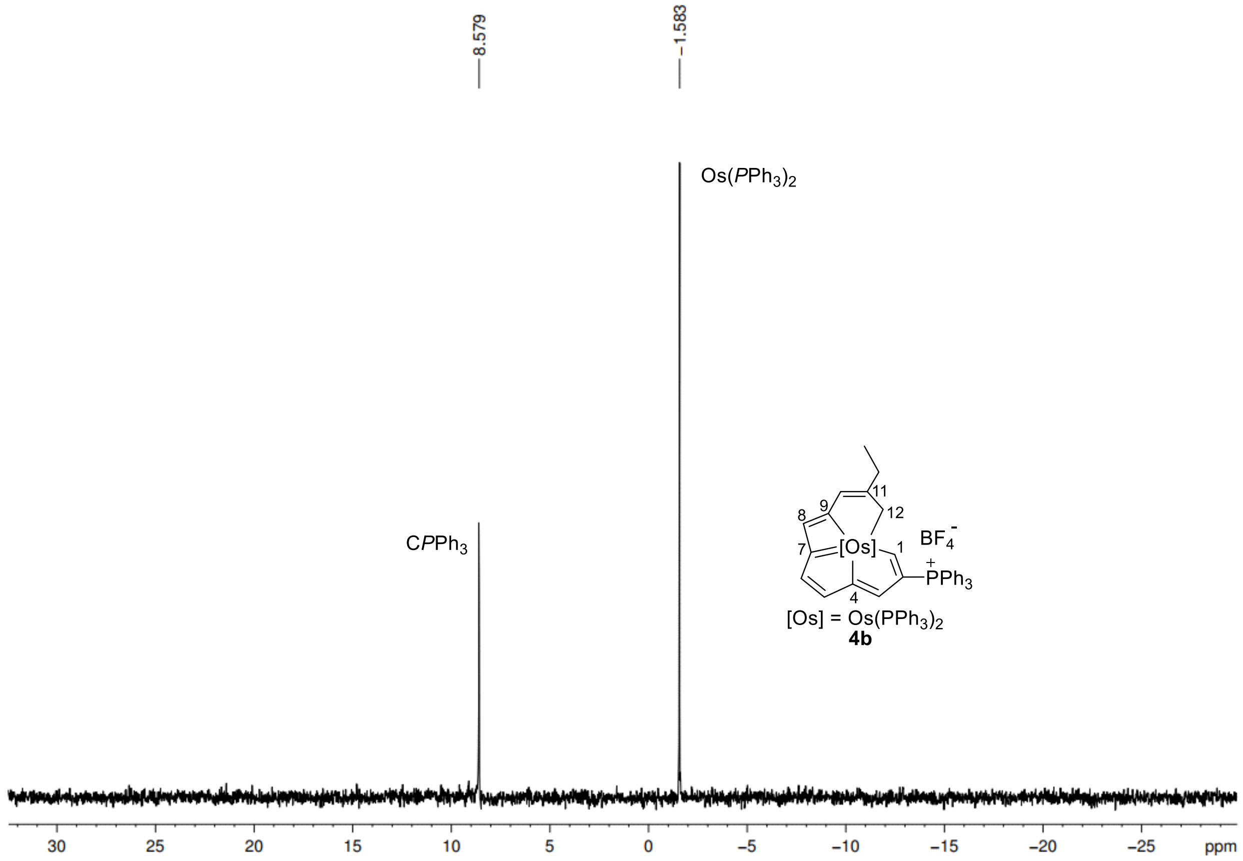

Figure S37. ^{31}P-NMR spectrum (242.9 MHz) of **4b** in CD_2Cl_2.

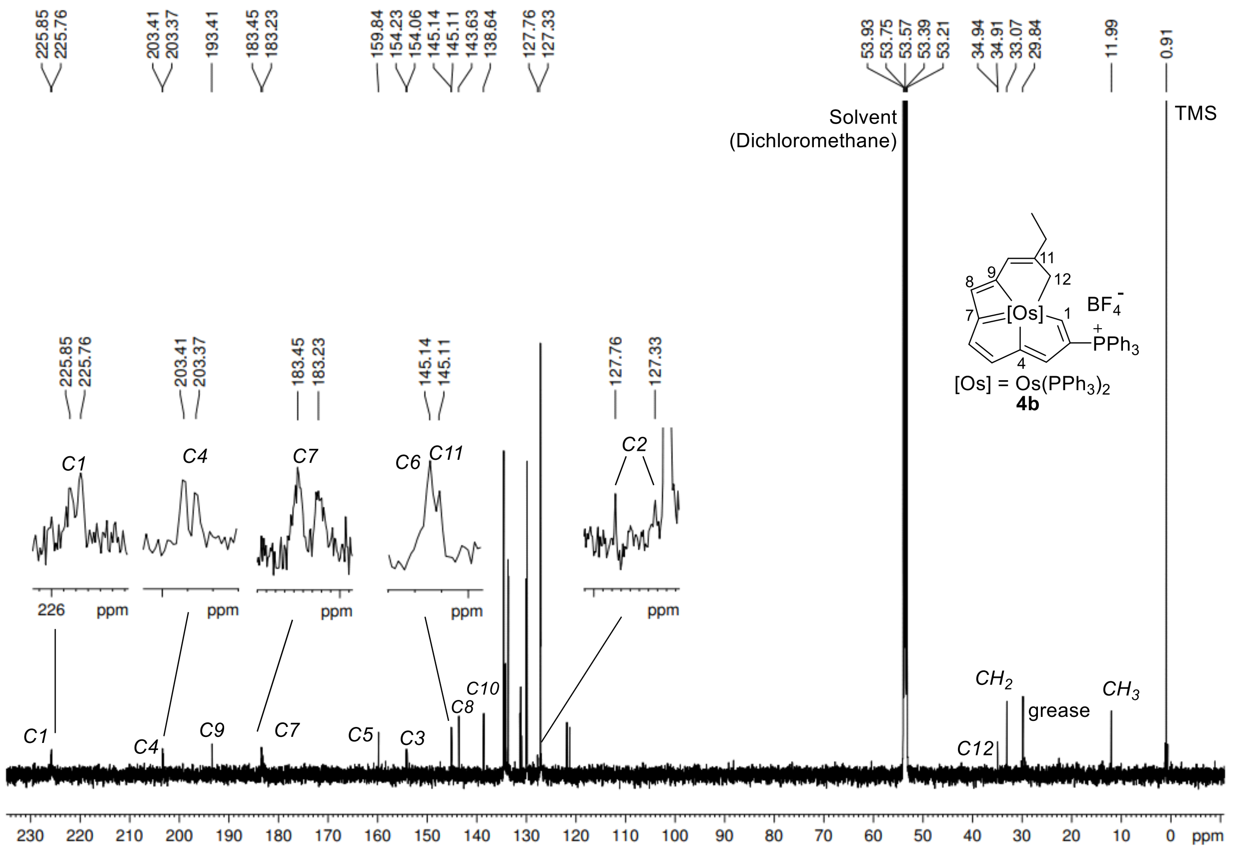

Figure S38. ^{13}C-NMR spectrum (150.9 MHz) of **4b** in CD_2Cl_2.

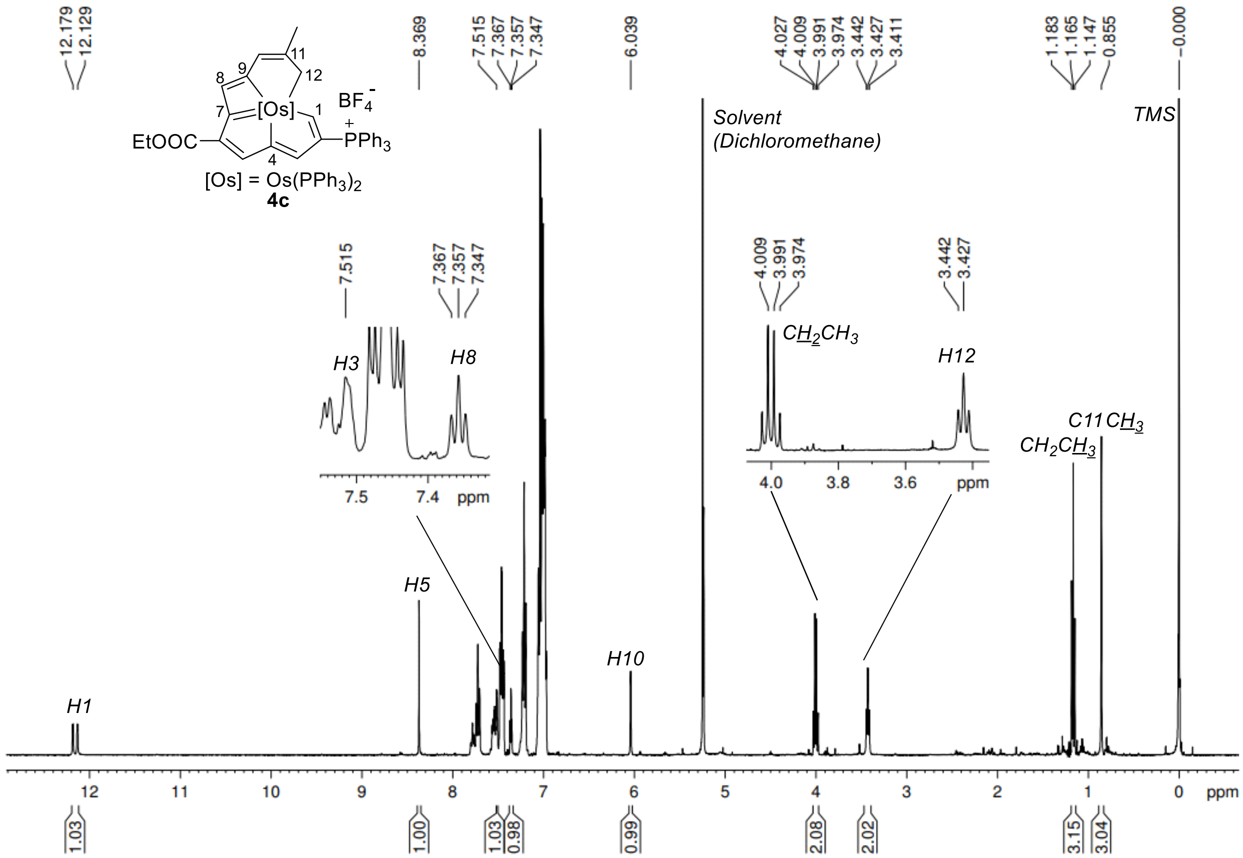

Figure S39. ^{1}H-NMR spectrum (600.1 MHz) of **4c** in CD_2Cl_2.

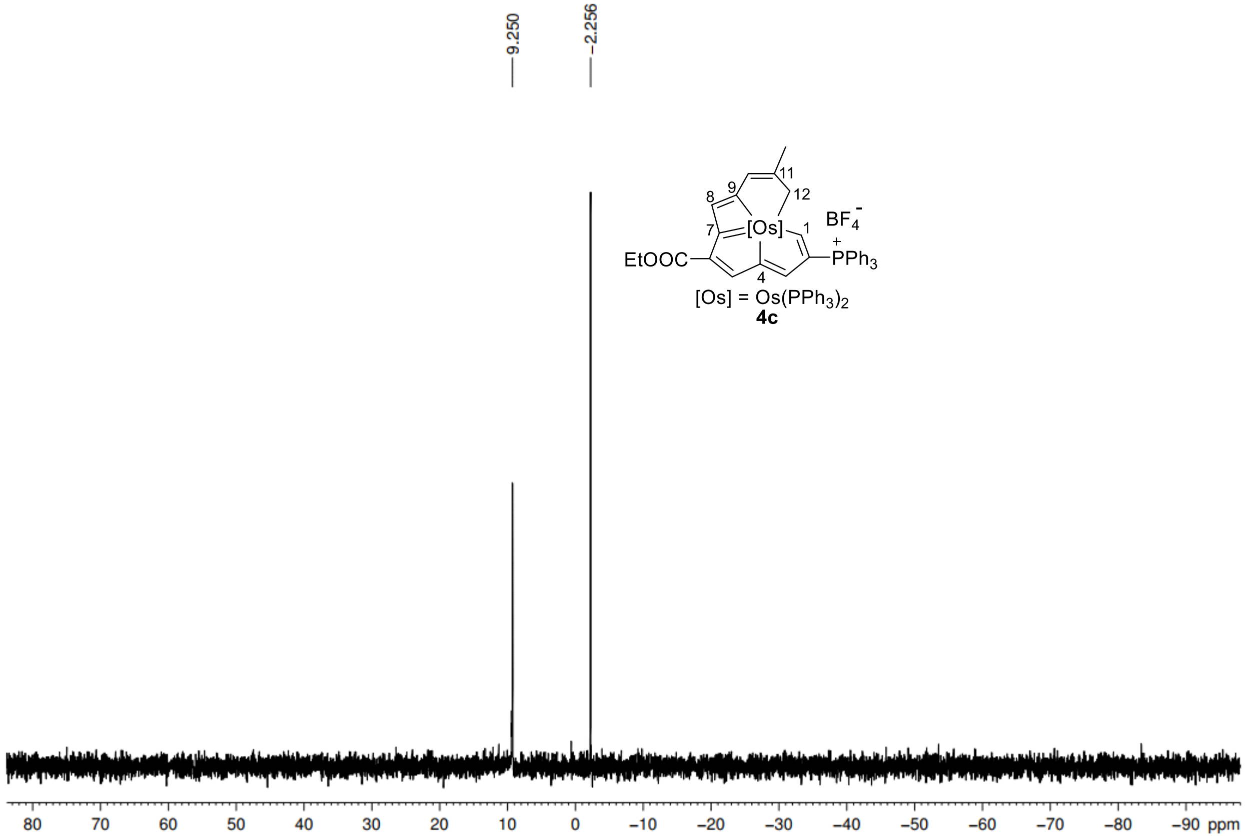

Figure S40. ^{31}P-NMR spectrum (242.9 MHz) of **4c** in CD_2Cl_2.

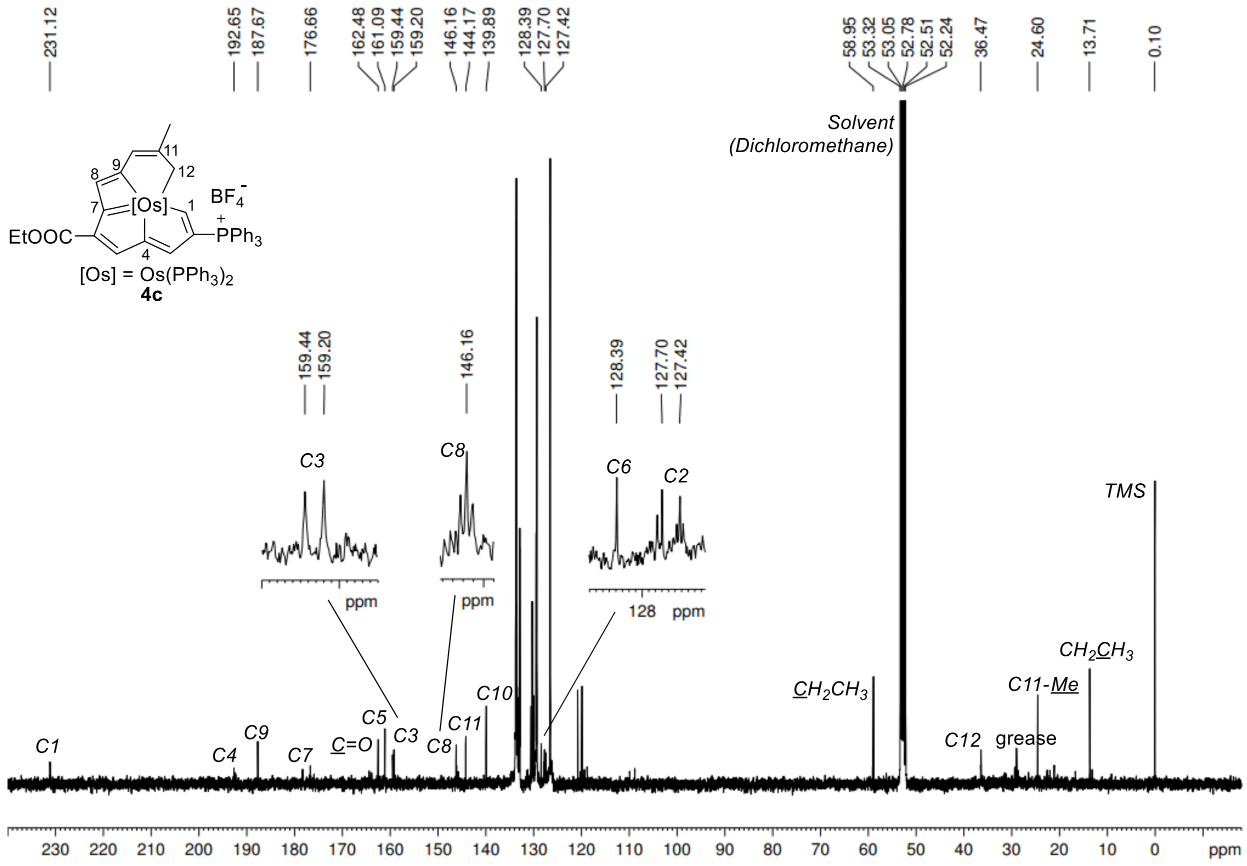

Figure S41. ^{13}C-NMR spectrum (151.9 MHz) of **4c** in CD_2Cl_2.

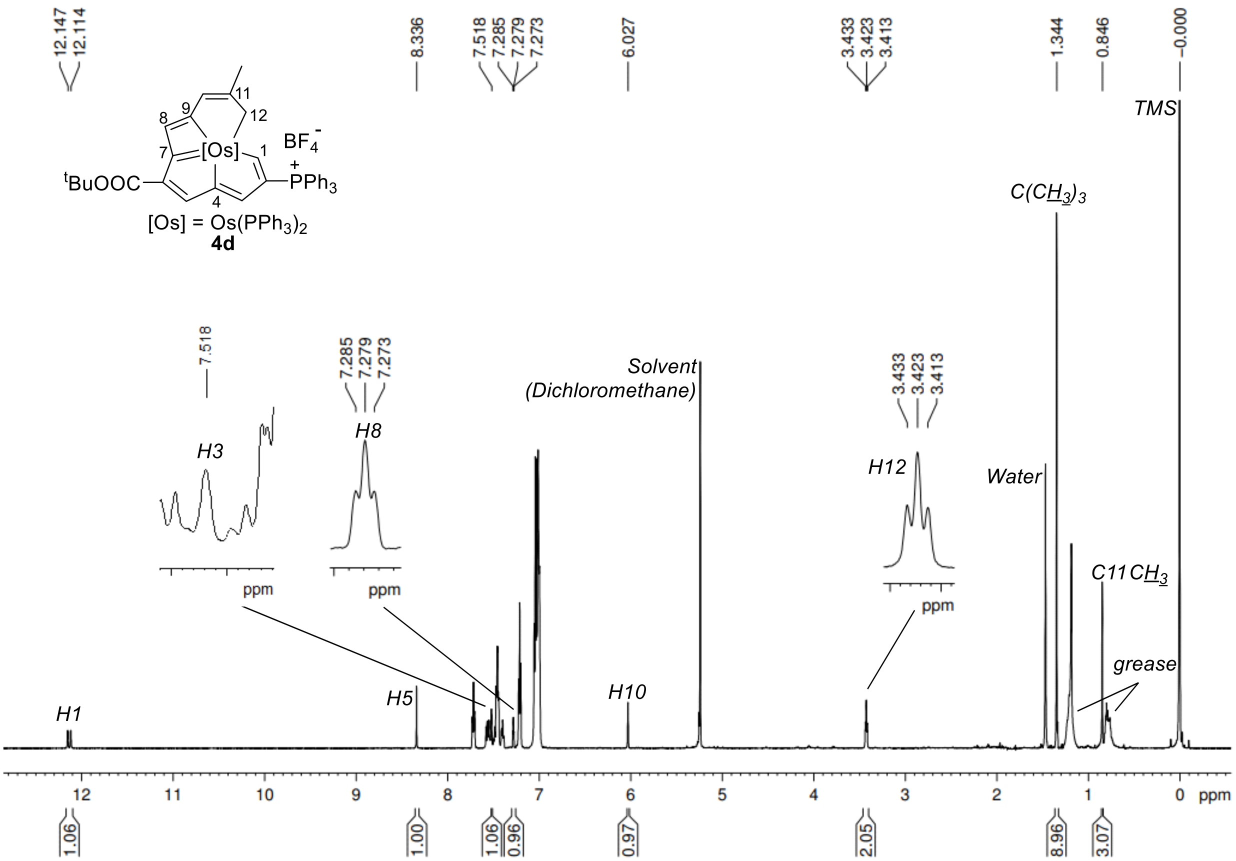

Figure S42. ^{1}H-NMR spectrum (600.1 MHz) of **4d** in CD_2Cl_2.

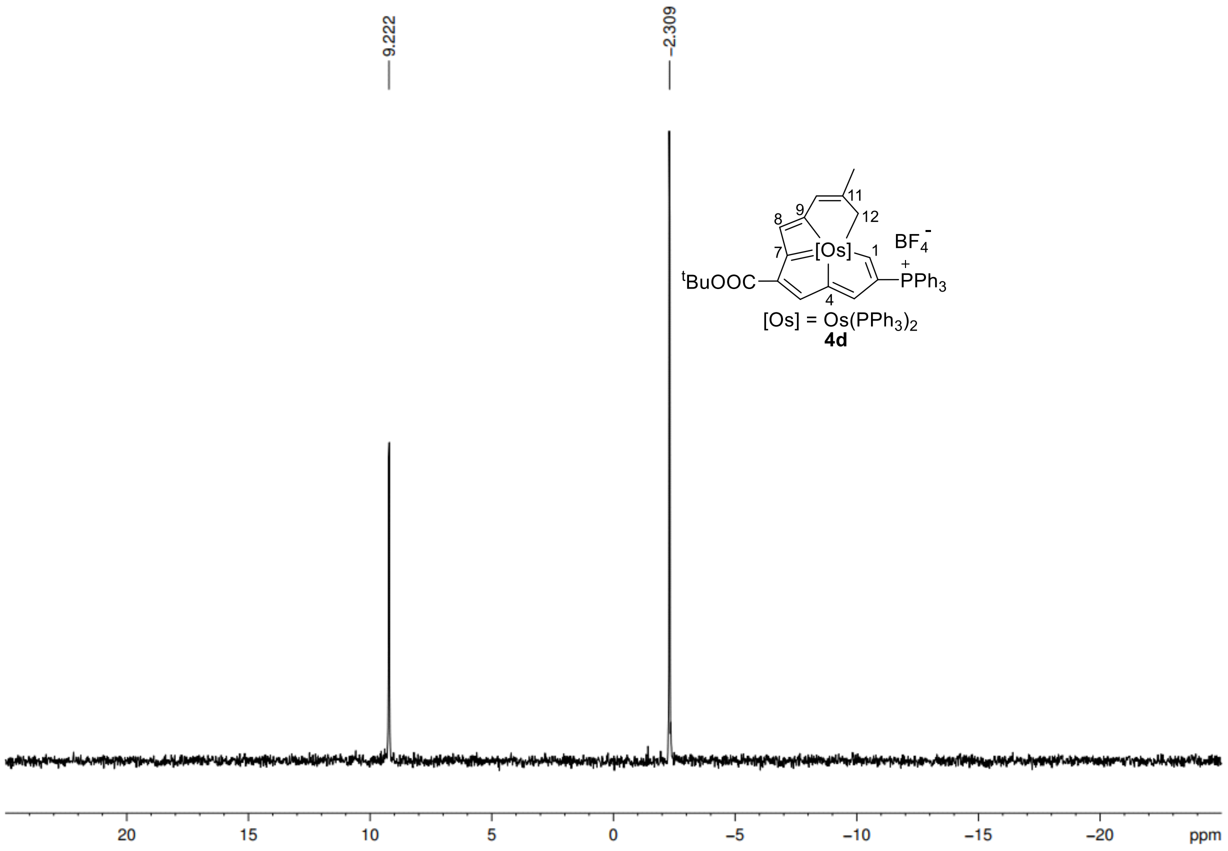

Figure S43. ^{31}P-NMR spectrum (242.9 MHz) of **4d** in CD_2Cl_2.

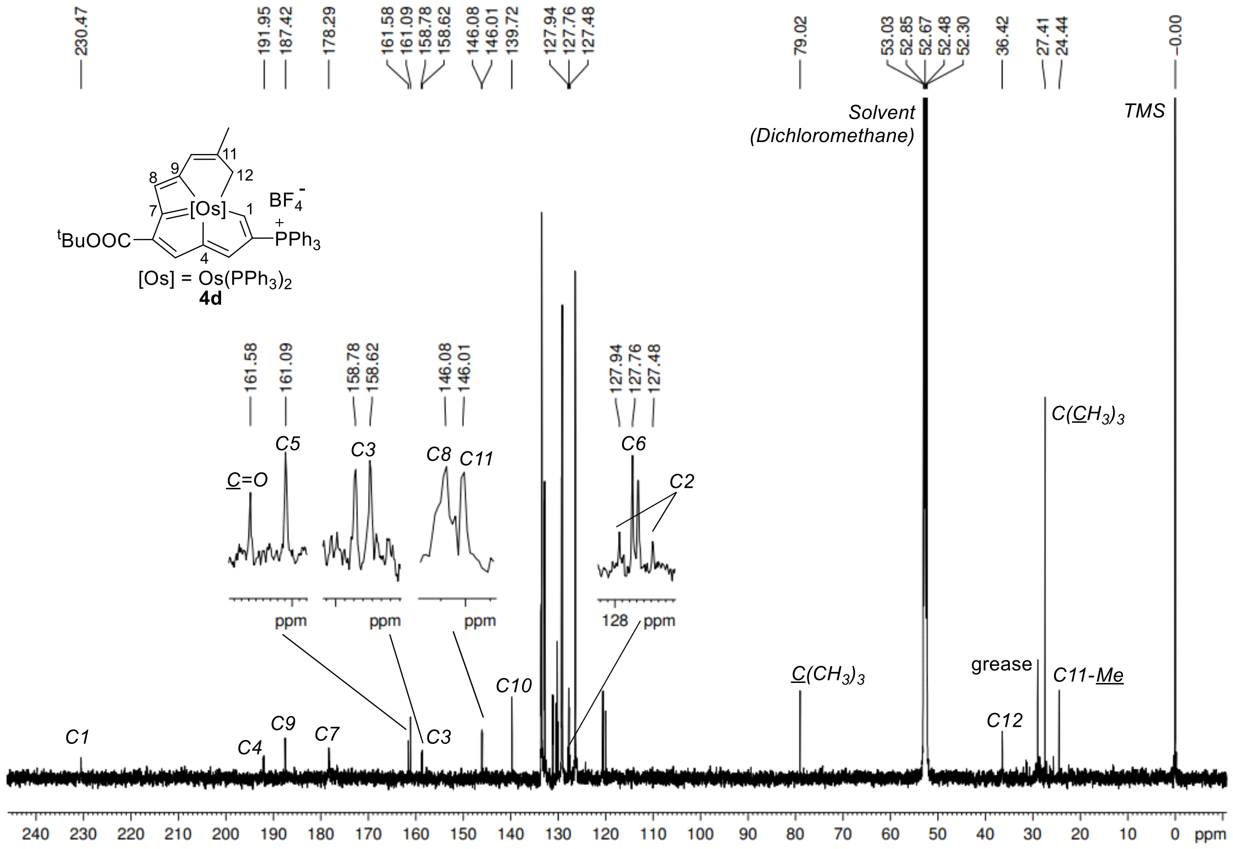

Figure S44. ^{13}C-NMR spectrum (150.9 MHz) of **4d** in CD_2Cl_2.

Table S4. Crystal data and structure refinement of **2**, **3a** and **3b**.

	**2**	**3a**	**3b**
Empirical formula	$C_{62}H_{53}B_2F_8OOsP_3$	$C_{73}H_{68}BF_4O_4OsP_3$	$C_{80}H_{65}BCl_5F_4O_2OsP_3$
Mol. weight	1270.77	1379.19	1605.49
Temperature [K]	100.01(10)	100.01(10)	100
Crystal system	monoclinic	monoclinic	monoclinic
Space group	$P2_1/n$	P21/c	Cc
a [Å]	12.71780(10)	17.4519(7)	30.6022(13)
b [Å]	21.6142(2)	17.5653(6)	13.7601(5)
c [Å]	19.3649(2)	21.0051(7)	19.7639(9)
α [°]	90	90	90
β [°]	96.4800(10)	105.479(4)	123.725(4)
γ [°]	90	90	90
V [Å^3]	5289.11(8)	6205.5(4)	6921.8(6)
Z	4	4	4
ρ_{calcd} [g cm^{-3}]	1.596	1.476	1.541
μ [mm^{-1}]	6.021	5.124	6.398
F(000)	2544	2800	3232
Crystal size [mm^3]	0.8 × 0.6 × 0.5	0.8 × 0.3 × 0.2	0.2 × 0.15 × 0.15
Radiation	Cu Kα (λ = 1.54184)	Cu Kα (λ = 1.54184)	CuKα (λ = 1.54178)
2θ range [°]	7.926 to 124.994	7.276 to 130	6.946 to 124.988
Coll. refl.	18135	21105	56322
Indep. refl.	8426	10382	10893
data/restraints/params	8426/157/731	10382/0/742	10893/14/866
GOF on F^2	1.112	1.084	1.051
R_1/wR_2 [$I \geq 2\sigma(I)$]	0.0439/0.1152	0.0651/0.1849	0.0443/0.1074
R_1/wR_2 (all data)	0.0449/0.1162	0.0671/0.1884	0.0500/0.1115
Largest peak/hole [e Å-3]	2.25/-3.62	2.14/-2.54	1.59/-0.95

Table S5. Crystal data and structure refinement of **4a**, **4b** and **5a**.

	**4a**	**4b**	**5a**
Empirical formula	$C_{67}H_{56}BF_4OsP_3$	$C_{68}H_{58}BF_4OsP_3$	$C_{70}H_{63}B_2F_8O_2OsP_3$
Mol. weight	1231.03	1245.06	1392.93
Temperature [K]	100.00(10)	100	100.01(10)
Crystal system	monoclinic	monoclinic	monoclinic
Space group	P21	Cc	P21/c
a [Å]	12.4255(2)	24.4508(9)	23.1230(5)
b [Å]	17.2338(2)	29.3023(10)	13.1857(3)
c [Å]	13.0213(2)	31.3585(11)	22.2830(5)
α [°]	90	90	90
β [°]	107.8160(10)	97.584(2)	101.246(2)
γ [°]	90	90	90
V [Å^3]	2654.64(7)	22270.7(14)	6663.5(3)
Z	2	16	4
ρ_{calcd} [g cm^{-3}]	1.54	1.485	1.388
μ [mm^{-1}]	5.856	5.591	4.841
F(000)	1240	10048	2808
Crystal size [mm^3]	0.534 × 0.358 × 0.17	0.1 × 0.1 × 0.05	0.1 × 0.1 × 0.1
Radiation	Cu Kα (λ = 1.54184)	CuKα (λ = 1.54178)	Cu Kα (λ = 1.54184)
2θ range [°]	7.13 to 129.984	4.732 to 136.756	7.756 to 130
Coll. refl.	32497	188281	24569
Indep. refl.	9057	37125	11355
data/restraints/params	9057/25/687	37125/174/2764	11355/384/837
GOF on F^2	1.03	1.032	1.041
R_1/wR_2 [$I \geq 2\sigma(I)$]	0.0397/0.1011	0.0424/0.0856	0.0609/0.1577
R_1/wR_2 (all data)	0.0432/0.1026	0.0723/0.0953	0.0697/0.1654
Largest peak/hole [e Å-3]	1.09/-1.29	1.22/-0.82	2.15/-1.54

5. Computational Cartesian Coordinates

3’

Os	-0.002192	-0.237982	0.003410
P	-0.055643	-0.350539	-2.321330
P	0.001197	-0.141431	2.334141
C	-2.155673	-0.202963	-0.024531
C	-0.594879	1.789230	-0.059802
C	-1.992997	2.118742	-0.122640
H	-2.336932	3.159699	-0.169118
C	2.882504	-1.182070	-0.038170
C	3.025257	0.221006	-0.050321
H	4.001227	0.723110	-0.098203
C	1.819382	0.914200	-0.018131
C	-2.486696	-1.498295	0.172874
C	1.571310	-1.624001	-0.001877
H	1.413769	-2.714459	0.013919
C	0.397017	2.787530	-0.029435
H	0.151908	3.857517	-0.032060
C	-2.841010	1.046268	-0.117504
C	1.703437	2.314253	-0.000763
H	2.576854	2.979961	0.031301
C	-1.160722	-2.153655	0.171583
H	-0.970073	-2.787063	-0.713383
H	-0.942902	-2.779940	1.056267
H	1.156233	-0.274238	-3.055874
H	-0.598647	-1.506350	-2.946228
H	-0.795341	0.623677	-3.040516
H	0.016207	1.127955	2.967526
H	-1.088946	-0.711117	3.047885
H	1.062015	-0.740833	3.061386
H	3.747467	-1.857214	-0.049633
H	-3.477835	-1.947626	0.359395
H	-3.933325	1.131609	-0.171698

4’

Os	0.038309	-0.148242	0.018156
P	-0.085701	0.197981	2.410865
P	0.242799	-0.549252	-2.365577
C	-0.092816	1.903879	-0.257071
C	-2.939721	-0.686149	-0.052265
H	-3.853506	-0.550618	-0.149067
C	1.248618	2.289766	-0.259169

H	1.643572	3.125995	-0.363493
C	3.111503	0.482539	0.087517
H	3.889271	0.992617	0.112094
C	1.803871	1.040765	-0.056517
C	-1.348329	2.564816	-0.379064
H	-1.444183	3.485574	-0.467904
C	-2.390781	1.692195	-0.347191
H	-3.276066	1.965723	-0.428947
C	-2.022299	0.311035	-0.177699
C	-2.362160	-1.966534	0.245531
C	3.091304	-0.862138	0.184007
C	1.750532	-1.532265	0.279091
H	1.675547	-1.962045	1.145728
H	1.697276	-2.224489	-0.398688
C	-0.963900	-1.939453	0.284049
H	-0.487270	-2.723540	0.427736
H	-2.940897	-2.849472	0.419845
H	3.997439	-1.431125	0.192531
H	0.875773	-0.527210	3.020916
H	0.095289	1.507177	2.686089
H	-1.297322	-0.192944	2.859919
H	-0.031953	-1.844437	-2.629214
H	-0.627366	0.239816	-3.030921
H	1.501517	-0.266297	-2.763151

5’

Os	-0.005612	-0.260026	0.016054
P	0.441258	-0.370123	-2.395007
P	-0.129982	0.025674	2.418920
C	1.711759	-1.463492	0.358372
H	1.630308	-2.381498	0.485860
C	2.917681	-0.871281	0.389013
C	-0.820892	1.685102	-0.121140
C	0.081080	2.764308	-0.214098
H	-0.203422	3.647982	-0.295701
C	2.907267	0.547642	0.179599
H	3.679165	1.066011	0.169352
C	1.386302	2.421881	-0.177435
H	2.063210	3.053593	-0.262518
C	1.661234	1.063290	-0.003577
C	-2.228083	1.923100	-0.118929
H	-2.601122	2.774039	-0.064134

C	-2.199878	-0.432197	-0.452212
H	-2.294301	-0.669064	-1.399036
C	-2.965928	0.784366	-0.205710
C	-0.948605	-2.378685	-0.143758
H	-1.017808	-2.739873	-1.041121
H	-0.622759	-3.031616	0.495247
C	-2.031600	-1.613907	0.280397
H	-4.030770	0.780900	-0.100838
H	-2.598575	-1.798584	1.168842
H	3.821176	-1.419163	0.557602
H	-0.066778	-1.516624	-2.894940
H	-0.137705	0.683843	-3.008566
H	1.774035	-0.338141	-2.607573
H	0.468242	-1.020105	3.027995
H	0.496604	1.169006	2.769177
H	-1.424090	0.087304	2.798378

II-Os’

Os	0.088476	-0.300042	-0.000019
P	0.242620	-0.247000	2.332170
P	0.241099	-0.246673	-2.332334
C	1.934763	-1.316684	-0.000200
H	2.065351	-2.409803	-0.000117
C	3.085014	-0.549365	-0.000429
C	2.897299	0.858543	-0.000486
H	3.739207	1.564355	-0.000643
C	1.570587	1.257982	-0.000320
C	1.081163	2.576150	-0.000154
H	1.739096	3.455884	-0.000267
C	-0.300666	2.687518	0.000214
H	-0.797654	3.668186	0.000405
C	-1.060332	1.485509	0.000353
C	-2.478556	1.632722	0.000659
H	-2.882593	2.654957	0.000870
C	-3.391073	0.602926	0.000656
C	-3.052995	-0.783409	0.000379
H	-3.865809	-1.519281	0.000393
C	-1.760620	-1.187368	0.000143
C	-0.857405	-2.263797	-0.000114
H	-0.733381	-2.866037	-0.911268
H	-4.455245	0.861991	0.000850
H	-0.733167	-2.866383	0.910776

H	-0.332084	-1.282931	3.118753
H	1.544498	-0.254960	2.891685
H	-0.285241	0.860378	3.044643
H	-0.336373	-1.281237	-3.118705
H	-0.285180	0.861945	-3.044055
H	1.542466	-0.256987	-2.893012
H	4.093058	-0.986085	-0.000531

6. References

(1). Zhu, C.; Zhou, X.; Xing, H.; An, K.; Zhu, J.; Xia, H. σ-Aromaticity in an unsaturated ring: osmapentalene derivatives containing a metallacyclopropene unit. *Angew. Chem., Int. Ed.* **2015**, *54*, 3102-3106.

(2). Huang, F.; Zheng, X.; Lin, X.; Ding, L.; Zhuo, Q.; Wen, T. B.; Zhang, H.; Xia, H. Extension of the Simmons-Smith reaction to metal-carbynes: Efficient synthesis of metallacyclopropenes with σ-aromaticity. *Chem. Sci.* **2020**, *11*, 10159-10166.

(3). Tomita, R.; Al-Maharik, N.; Rodil, A.; Bühla, M.; O'Hagan, D. Synthesis of aryl α,α-difluoroethyl thioethers a novel structure motif in organic chemistry, and extending to aryl α,α-difluoro oxyethers. *Org. Biomol. Chem.* **2018**, *16*, 1113-1117.

(4). Bertrand, M.; Monti, H. Sur une synthese stereospecifique des α-cyclopropylcetones substituees. acetyl-1 dimethyl-1,2 cyclopropanes cis et trans. *Tetrahedron Lett.* **1968**, *9*, 1069-1073.

(5). Becke, A. D. Density-functional thermochemistry. III. The role of exact exchange. *J. Chem. Phys.* **1993**, *98*, 5648-5652.

(6). Zhao, Y.; Truhlar, D. G. A new local density functional for main-group thermochemistry, transition metal bonding, thermochemical kinetics, and noncovalent interactions. *J. Chem. Phys.* **2006**, *125*, 194101.

(7). Frisch, M. J. *et al.* Gaussian 16, Revision A.01, Gaussian, Inc., Wallingford CT, **2020**.

(8). Jiao, H.; Schleyer, P. v. R.; Mo, Y.; McAllister, M. A.; Tidwell, T. T. Magnetic evidence for the aromaticity and antiaromaticity of charged fluorenyl, indenyl, and cyclopentadienyl systems. *J. Am. Chem. Soc.* **1997**, *119*, 7075-7083.

(9). Geuenich, D.; Hess, K.; Köhler, F.; Herges, R. Anisotropy of the induced current density (ACID), a general method to quantify and visualize electronic delocalization. *Chem. Rev.* **2005**, *105*, 3758-3774.

(10). Glendening, E. D. *et al.* NBO 7.0. Theoretical Chemistry Institute, University of Wisconsin, Madison, WI, **2018**.

(11). Lu, T.; Chen, F. Multiwfn: A multifunctional wavefunction analyzer. *J. Comput. Chem.* **2012**, *33*, 580-592.

(12). Dolomanov, O. V.; Bourhis, L. J.; Gildea, R. J.; Howard, J. A. K.; Puschmann, H. OLEX2: A complete structure solution, refinement and analysis program. *J. Appl. Cryst.* **2009**, *42*, 339.

(13). Sheldrick, G. M. SHELXT– Integrated space-group and crystal-structure determination. *Acta Cryst. A* **2015**, *A71*, 3-8.

(14). Sheldrick, G. M. Crystal structure refinement with SHELXL. *Acta Cryst. C* **2015**, *C71*, 3-8.
